# Scanning Electron Microscopy and Triple TOF-LC-MS-MS Analysis of Polyphenols from PEF-Treated Edible Mushrooms (*L. edodes*, *A. brunnescens*, and *P. ostreatus*)

**DOI:** 10.3390/antiox12122080

**Published:** 2023-12-06

**Authors:** Mara Calleja-Gómez, Patricia Roig, Suzana Rimac Brnčić, Francisco J. Barba, Juan Manuel Castagnini

**Affiliations:** 1 Research Group in Innovative Technologies for Sustainable Food (ALISOST), Department of Preventive Medicine and Public Health, Food Science, Toxicology and Forensic Medicine, Faculty of Pharmacy and Food Sciences, Universitat de València, Avda. Vicent Andrés Estellés s/n, Burjassot, 46100 València, Spain; mara.calleja@uv.es (M.C.-G.); patricia.roig@uv.es (P.R.); juan.castagnini@uv.es (J.M.C.); 2 Faculty of Food Technology and Biotechnology, University of Zagreb, Pierottiejva 6, 10000 Zagreb, Croatia; srimac@pbf.hr

**Keywords:** pulsed electric fields, recovery, antioxidant compounds, phenolic profile, bioactive, mushroom, scanning electron microscopy

## Abstract

Pulsed electric fields (PEF) technology has been used as a sustainable method for extracting antioxidant bioactive compounds from different food matrices. In the present study, the optimal conditions of PEF extraction for mushrooms (2.5 kV/cm, 50 kJ/kg, 6 h) were applied to *Lentinula edodes*, *Agaricus brunnescens*, and *Pleurotus ostreatus* to evaluate the total antioxidant capacity of the extracts, followed by the Triple TOF-LC-MS-MS analysis of the phenolic profile compared to *A. bisporus* by high-performance liquid chromatography coupled to mass spectrophotometry. In addition, the microporation effect of the technology on the mushroom surface was evaluated using scanning electron microscopy. A comparison was made with a maceration extraction (aqueous stirring for 6 h). The results showed that PEF-assisted extraction enhanced the recovery of antioxidant compounds such as 3,5-dicaffeoylquinic and cinnamic acid with contents up to 236.85 µg/100 g dry weight and 2043.26 µg/100 g dry weight from *A. bisporus*, respectively. However, mixed results were obtained for certain phenolic compounds, including vanillic acid from *L. edodes*, ellagic acid from *P. ostreatus*, and thymol from all mushrooms. These results indicate that the application of PEF technology is effective for the extraction of antioxidant compounds in fungal matrices by creating micropores in cell membranes that allow great recovery in matrices with high content of bioactive compounds.

## 1. Introduction

Oxidation processes are normal in many organisms due to cellular metabolism. However, the excessive production of oxygen-free radicals can lead to pathological processes due to cellular damage, causing cardiovascular diseases, cancer, inflammatory disorders, and neurodegenerative diseases, among others [1,2]. For this reason, in recent years, the effect of several antioxidant compounds that protect against free radicals through their capacity to neutralize them has been studied in vegetable matrices, seeds, wines, and fruits [3,4].

One of these matrices are the mushrooms that have been consumed for decades in Asian countries for their therapeutic and gastronomic properties and whose production and consumption is now globalized. In this sense, the per capita consumption of mushrooms has quadrupled since 1997 (from 1 kg in 1997 to 4.7 kg), accompanied by an increase in the production of cultivable mushrooms, led by China [5]. According to the article published by Singh et al. [6] and shared by the Food and Agriculture Organization of the United Nations (FAO), mushroom production in 2019 amounted to 43 million tons, with *Lentinula edodes* (26%), *Pleurotus ostreatus* (16%), and *Agaricus bisporus* (11%) making a greater contribution and expanding their cultivation in Europe, America, and Canada. In Europe, the growing production of mushrooms is driven by consumer demand for healthy foods with a good nutritional profile that can be integrated into vegetarian and vegan diets [7]. This trend is accompanied by an increase in innovative technologies that allow mushrooms to be used not only as food but also for compost production, making Europe the continent with the highest market growth in recent years [8].

Regarding their nutritional quality, mushrooms are not only a great source of bioactive molecules but also have a high antioxidant capacity due to a variety of vitamins (C and D), selenium, and phenolic compounds. These phenolic compounds, especially phenolic acids, are found in a wide variety of fungal species, including the most commonly consumed mushrooms in the world: *Agaricus bisporus* (white mushroom), *Pleurotus ostreatus* (oyster mushroom), and *Lentinula edodes* (shiitake mushroom) [9]. Several studies [10,11,12] have shown that consumption of mushrooms reduces oxidative stress and has a protective effect against different diseases. However, not all mushrooms have the same antioxidant capacity, which varies depending on the species and growing conditions. In this sense, this antioxidant capacity is due mainly to the presence of gallic acid and, to a lesser extent, vanillic acid, depending on the phenolic profile of the mushroom species. The presence of flavonoids in mushrooms has been strongly questioned, suggesting that they cannot synthesize flavonoids, although fungi share similarities with plants in terms of biosynthetic pathways to generate phenolic compounds, such as melanin precursors [13].

Consequently, the extraction of antioxidant bioactive compounds from mushrooms using innovative techniques that allow higher recovery of these compounds to avoid the thermal degradation of conventional methods at high temperatures is of interest [14]. Pulsed electric fields (PEF) technology is an innovative methodology used to improve the extraction of nutrients from different plant matrices, as it offers advantages over conventional methods: short extraction times, low temperatures, use of non-toxic solvents, and energy saving [15,16]. In this way, the impact on the environment is reduced, and the thermosensitive components are preserved by electroporation of the cell membranes, allowing for selective recovery. The application of PEF in the extraction of bioactive compounds has been extensively studied in recent years. Positive results have been obtained in the recovery of various compounds, including polyphenols, pigments, and macronutrients, from mixed matrices [17,18,19]. To the best of our knowledge, in addition to many research studies, no comprehensive study was performed on the characterization of different edible mushrooms in terms of antioxidant capacity and specific phenolic compounds, the differences in both PEF methodology and type of mushroom, and, in particular, the different behavior of specific compounds towards the same pretreatment and the need for individual optimization in relation to the compound of interest. We also noted that most publications indicate a global increase in antioxidant capacity without considering how the methodology applied affects a specific target.

For the aforementioned, the present study aims to evaluate whether PEF extraction improves the extraction of phenolic compounds with antioxidant capacity compared to conventional aqueous extraction from the edible mushrooms *L. edodes*, *A. bisporus*, *A. brunnescens*, and *P. ostreatus*, analyzing the differences in the enhancement of extraction in relation to the polyphenol obtained. Moreover, the correlation between the amount of extracted antioxidant compounds and the fungal surface cell membrane electroporation after PEF treatment by scanning electron microscopy (SEM) was also evaluated.

## 2. Materials and Methods

### 2.1. Chemical Reagents

The reagents 2,2′-azinobis (3-ethylbenzothiazoline-6-sulfonic acid) (ABTS, ≥98%), 2,2′-azinobis (2-methylpropanimidamide) dihydrochloride) (AAPH, 97%), 6-hydroxy-2,5,7,8-tetramethylchroman-2-carboxylic acid (Trolox, ≥98%), gallic acid (C_7_H_6_O_5_, ACS reagent, ≥98.0%), potassium persulfate (K_2_S_2_O_8_, ACS reagent, ≥99.0%), Folin–Ciocalteu reagent (2 M), and fluorescein sodium salt (C_20_H_10_Na_2_O_5_) were purchased from Sigma-Aldrich (Steinheim, Baden-Wuerttemberg, Germany). Sodium carbonate (Na_2_CO_3_, 98%) was obtained from VWR (Saint-Prix, France), and both disodium phosphate (Na_2_HPO_4_, 99%) and potassium dihydrogen phosphate (KH_2_PO_4_, ACS 99%) were purchased from VWR International Eurolab (Barcelona, Spain). Ultrapure water was obtained from Milli-Q SP Reagent Water System (Millipore Corporation, Bedford, MA, USA).

### 2.2. Sample Preparation

*Agaricus bisporus* (white button mushroom), *Lentinula edodes* (shiitake mushroom), *Agaricus brunnescens* (portobello mushroom), and *Pleurotus ostreatus* (oyster mushroom) were purchased from a local supermarket (València, Spain). They were stored under refrigeration at 4 °C for a maximum of 24 h until use. The samples were cut into 3 × 5 mm slices with a knife, using the pileus for the extractions, until a total of 20 g was reached for each sample. To observe the microstructure by SEM, the resulting solid was taken after the extractions and subjected to a freeze-drying process. The moisture content (g water/100 g sample) was 0.88 ± 0.01 for *A. bisporus*, 0.92 ± 0.01 for *L. edodes*, and 0.91 ± 0.02 for *A. brunnescens* and *P. ostreatus*, respectively.

### 2.3. Pulsed Electric Field-Assisted Extraction

Mushroom pretreatment was performed with a PEF-Cellcrack III (German Institute of Food Technologies (DIL)) equipment (ELEA, Quakenbrueck, Germany) at the Faculty of Pharmacy, University of València (Burjassot, Valencia, Spain). In each case, 20 g of the fresh sliced mushrooms were added to a 900 mL vessel together with 200 mL water (1:1 *v*/*v* tap water and distilled water) until a conductivity of 800 µS/cm, measured with the ProfiLine Cond 3310 conductometer (WTW, Xylem Analytics, Weilheim in Oberbayern, Germany), was reached. The optimal conditions were obtained after preliminary research with PEF based on the study published by Calleja-Gómez et al. [15] for the extraction of bioactive compounds and nutrients from *A. bisporus* (50 kJ/kg specific energy, 2.5 kV/cm electric field and 6 h total extraction time) were applied with a pulse frequency and duration of 2.00 Hz and 100 ms, respectively.

The PEF pretreatment took 20 s; after that, the conductivity and temperature of the extracts were measured again, and the extracts were shaken at a room temperature for 6 h. Then, the extracts were filtered and centrifuged (4000 rpm, 15 min) to separate the liquid fraction from the solid residue and were stored frozen at −20 °C until use. The solid residue of each extract was subjected to freeze-drying and stored in the freezer at the same temperature. All extracts obtained by PEF were compared with those obtained by conventional extraction (maceration), in which they were kept in aqueous solution under stirring without pretreatment for 6 h at room temperature. Two replicates were performed for each sample and method.

### 2.4. Chemical Analysis

#### 2.4.1. Total Antioxidant Capacity

The Trolox equivalent antioxidant capacity (TEAC) assay was performed according to the study published by Miller et al. [20]. It is based on the absorbance of the ABTS·+ radical at 734 nm, where the antioxidant compounds in the sample neutralize the ABTS·+, resulting in a decrease in absorbance. To perform the assay, 440 µL of potassium persulfate (140 mM) was added to 25 mL of ABTS (7 mM), and the solution was incubated at 20 °C in the dark for 18 h until the working solution was formed. The solution was mixed with 99% ethanol until an absorbance in the range of 0.68–0.72 was obtained. A cuvette containing 2 mL ethanol and 100 µL Trolox (standard)/sample was then filled, using various concentrations of Trolox between 0 and 250 µM, and measured on a Perkin-Elmer UV/Vis Lambda 2 spectrophotometer (Perkin-Elmer, Juegesheim, Germany).

The oxygen radical absorbance capacity (ORAC) assay was performed following the study published by Cao et al. [21]. The antioxidant compounds in the studied sample retard the oxidative degradation of fluorescein and consequently the decrease in absorbance over time upon the addition of the oxidizing reagent AAPH. Trolox (1 mM) was used as a standard and phosphate buffer (pH 7.2) as a blank. In a 96-well microplate, 50 µL blank/Trolox/sample was added along with 50 µL fluorescein and incubated at 37 °C for 10 min. Finally, 25 µL of AAPH was added to start the reaction. Successive measurements were performed every minute for one hour at 520 nm emission and 480 nm excitation wavelengths using a FLUOstar OMEGA plate reader (BMG Labtech GmbH, Ortenberg, Germany). One ORAC unit indicates that the antioxidant capacity of the sample is equivalent to 1 µM Trolox.

Results for both assays were expressed as µmol Trolox equivalents/g dry weight (µmol TE /g DW).

#### 2.4.2. Polyphenol Analyses

Total phenolic content (TPC) was determined by the Folin–Ciocalteu method described by Singleton et al. [22], which is based on the formation of blue-colored phosphomolybdenum and phosphotungsten complexes in a basic medium after reaction with the phenols in the sample. For the determination, 3 mL Na_2_CO_3_, 100 µL Folin–Ciocalteu reagent (50% *v*/*v*), and 100 µL gallic acid standard/sample were added to each tube. The tubes were incubated at room temperature for one hour, and, subsequently, absorbance was measured at 750 nm using a Perkin-Elmer UV/Vis Lambda 2 spectrophotometer (Perkin-Elmer, Juegesheim, Germany). Results were expressed as mg gallic acid equivalents/g dry weight (mg GAE/g DW).

The characterization and quantification of polyphenols were performed by Triple TOF-LC-MS-MS according to the method described in the study published by Roselló-Soto et al. [23]. The separation of the different phenolic compounds was performed using a Waters C18 column 1.7 µm (2. 1 × 50 mm) Acquity UPLC BEH.C18 (Waters, Cerdanyola del Vallès, Spain) implemented on an Agilent 1260 Infinity chromatography instrument (Agilent, Waldbronn, Germany). Subsequent compound identification was performed on a TripleTOF™ 5600 LC/MS/MS system (AB SCIEX, Foster City, CA, USA). For HPLC separation, a mobile phase consisting of two solvents was used: A (0.1% formic acid, water) and B (0.1% formic acid, methanol), with a flow rate of 0.4 mL/min and an injection volume of 5 µL of the sample. MS data were obtained in the range of 80–1200 *m*/*z* in negative mode, and the acquisition method was carried out by survey scan type (TOF-MS).

PeakView1.1 software (AB SCIEX, Foster City, CA, USA) was used for data acquisition and analysis. External calibration and quantification were performed using a representative polyphenol from each group: Flavonoids (catechin (≥99.0%), naringenin (≥95.0%), kaempferol (≥99.0%), apigenin (≥95.0%), phenolic acids (gallic acid (≥99.0%), caffeic acid (≥98.0%), chlorogenic acid (≥99.0%), cinnamic acid (≥98.0%), vanillic acid (≥99.0%), ellagic acid (≥95.0%)), isoflavonoids (genistein (≥97%)), stilbenes (resveratrol (≥98.0)), phenylethanoids (hydroxytyrosol (≥98.0)), and terpenes (thymol (≥99.0%)). The compounds identified and quantified in some extracts are shown in Table S1 and the IDA survey in Figure S1. Concentrations of 0.25 to 2 ppm of the standards of interest were used to establish the calibration, as shown in Figure S4 (Supplementary material). All the standards calibration curves showed a ≥0.9990 linearity.

### 2.5. Scanning Electron Microscopy

A Hitachi S-4800 equipment (Servei Central de Suport a la Investigació Experimental—SCSIE, Universitat de València, Valencia, Spain) with ×300 magnification was used to evaluate the fungal surface. Samples were collected from both the freeze-dried sample (control sample) and the pre-treated and freeze-dried samples with conventional treatment and PEF. The samples were placed on a carbon sheet and metallized with a coating of Au and Pd for 2 min. Then, the difference on the surface of the mushrooms was determined to evaluate the electroporation produced by the electrical pulses.

### 2.6. Statistical Analysis

For data processing, analysis of variance (ANOVA) considering a statistical difference of *p* < 0.05 was used to determine the difference between PEF and conventional treatment of the samples. All statistical analyses were performed using GraphPad Prism 8.0.2 software (GraphPad Software, San Diego, CA, USA). All analyses were performed in triplicate. Standard deviations are represented by error bars in the figures. The quantification of phenolic compounds was carried out considering a relative standard deviation (RSD%) of a maximum of 10%.

## 3. Results and Discussion

### 3.1. Extraction of Total Antioxidant Compounds under PEF Optimal Conditions

**Figure 1:**
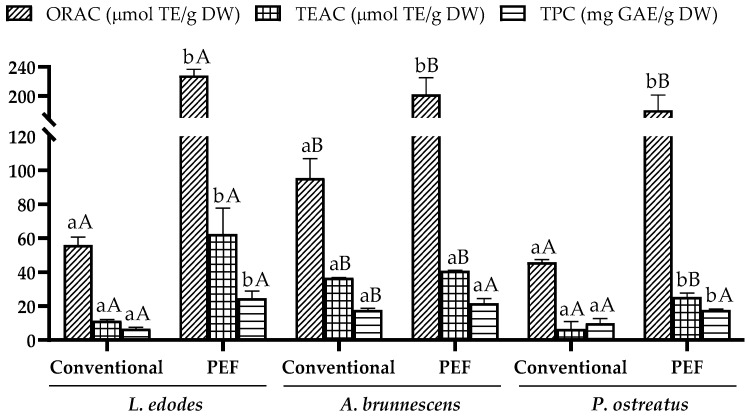
Oxygen radical absorbance capacity (ORAC) (µmol Trolox equivalents (TE)/g dry weight (DW)), Trolox equivalent antioxidant capacity (TEAC) (µmol Trolox equivalents (TE)/g dry weight (DW)) and total phenolic content (mg gallic acid equivalents (GAE)/g dry weight (DW)) values in pulsed electric field (PEF) and conventional extraction of *L. edodes*, *A. brunnescens* and *P. ostreatus*. Different lowercase letters indicate significant differences (*p* < 0.05) between extraction methodologies. Different capital letters indicate significant differences (*p* < 0.05) between mushrooms.

The results on the antioxidant capacity of the samples of *L. edodes*, *A. brunnescens*, and *P. ostreatus* are shown in Figure 1. The values ranged from 45.99 ± 1.44 to 228.07 ± 8.58 µmol TE/g DW for ORAC, 6.67 ± 2.38 to 62.50 ± 10.15 µmol TE/g DW for TEAC, and 6.71 ± 0.90 to 24.68 ± 4.19 mg GAE/g DW for TPC, corresponding to conventional and PEF-assisted extraction.

Regarding the type of mushroom, significant differences were observed in the three assays performed. The highest results in conventional extraction were obtained from *A. brunnescens* with 95.44 ± 11.48 µmol TE/g DW, 36.71 ± 0.15 µmol TE/g DW, and 17.86 ± 0.84 mg GAE/g DW for ORAC, TEAC, and TPC, respectively, which showed significant differences (*p* < 0.05) compared to the other mushrooms. However, the recovery of bioactive compounds by conventional extraction was lower in *L. edodes* and *P. ostreatus*, with no significant difference (*p* > 0.05). On the other hand, a significant difference (*p* < 0.05) was observed in the recovery of antioxidant compounds depending on the extraction method used, with greater recovery obtained using PEF in all the mushrooms studied. The highest values were obtained after applying PEF technology in *L. edodes* with 228.07 ± 8.58 µmol TE/g DW for ORAC, 62.50 ± 10.15 µmol TE/g DW for TEAC, and 24.68 ± 4.19 mg GAE/g DW for TPC.

In this sense, the increases obtained when comparing the two methods for all mushrooms are remarkable, especially for *L. edodes*, for which the PEF pretreatment was very effective with an increase in extraction of 307.14% for ORAC, 443.98% for TEAC, and 267.81% for TPC compared to conventional extraction. Moreover, the highest increase was observed for *A. brunnescens* in ORAC with 111.84% compared to conventional extraction and for *P. ostreatus* in TEAC with 279.76%. According to the obtained results, PEF technology improves the recovery of antioxidant compounds of high interest due to electroporation, which allows for selective extraction without high temperatures, avoiding the thermal degradation of thermosensitive antioxidant molecules. The improvements in antioxidant capacities of PEF extracts obtained from various plant and animal matrices have been reported in several studies. In the study published by Redondo et al. [24] on peach by-products, the use of PEF with water as solvent is very effective and increases the total bioactive compounds and phenolic compounds, among which phenolic acids stand out. This fact is also observed in animal matrices such as fish side streams [25], where aqueous extraction with PEF increased antioxidant capacities by 21.74% and 29.11% (TEAC) and by 22.11% and 40.93% (ORAC) for head and skin, respectively.

As can be seen in Table 1, emerging technologies achieved favorable results in the recovery of compounds with antioxidant capacity. According to the results published by Xue et al. [26], the pulsed electric fields technology in mushrooms not only increases the yield of polyphenols and antioxidant compounds by up to 50.9% compared to conventional methods but also could be a commercially applicable technique because it is an energy-efficient extraction due to the low electrical conductivity of the matrix, which minimizes ohmic heating. Compared to conventional extraction using various solvents, the extracts obtained by PEF showed a higher total phenolic content than conventional aqueous, methanolic, and ethanolic extraction. These results showed that the recovery of phenolic compounds depends not only on the technology but also on the solvent used: ethanol > water > methanol. Although ethanol extraction greatly enhances the recovery of phenolic compounds in mushrooms [27], the use of methods that increase cell membrane permeability, such as PEF, allows for greater extractions of these compounds by using aqueous solvents and avoiding the use of organic solvents or other processing steps, such as solvent evaporation for use in in vitro assays.

In this sense, the possible combination of non-thermal techniques for the extraction and preservation of these compounds in mushrooms is interesting, such as the combination with ultrasound (USN), which has been shown to increase the recovery of high-quality compounds in mushrooms [28], other food matrices [29], and by-products [30,31]. Ultrasound-assisted extraction (UAE) improves the production of target compounds, avoiding the use of organic solvents and shortening the extraction time. Thus, the combination with PEF acts as a synergy in recovery of target compounds due to the combined effect of cavitation and electroporation. Therefore, at the industrial level, it is of special interest to consider these combinations that effectively and quickly allow the application of a pre-treatment that improves extraction rates [32].

**Table 1:** Overview of the antioxidant capacities of extracts obtained by conventional and emerging methodologies from different mushroom species.

Extraction	Mushroom	Treatment	ORAC	TEAC	TPC	Ref.
Conv. (Water)	*Lentinula edodes* *Pleurotus ostreatus* *Agaricus brunnescens*	200 mL water, 25 °C, 6 h stirring	56.1 µmol TE/g DW 45.99 µmol TE/g DW 95.44 µmol TE/g DW	11.5 µmol TE/g DW 6.67 µmol TE/g DW 36.71 µmol TE/g DW	6.71 mg GAE/g DW 9.99 mg GAE/g DW 17.86 mg GAE/g DW	Present study
	*Agaricus bisporus*	200 mL, 9% (*w*/*w*), 95 °C, 1 h	-	-	1.3 mg GAE/g mushroom	[26]
	*Lentinula edodes*	1 L, 80% (*v*/*v*), 80°C, 30 min	-	290 µmol/g DW	-	[33]
Conv. (Methanol)	*Morchella elata*	300 mL (1:3 *w*/*v*) 3500 r/min, 30 min + 24 h	-	240 µmol TE/g extract	0.46 mg GAE/g DW	[34]
	*Suillus luteus*		-	154 µmol TE/g extract	0.67 mg GAE/g DW	
	*Pleurotus eryngii*		-	67.0 µmol TE/g extract	0.180 mg GAE/g DW	
	*Cyttaria gunnii*		-	77.0 µmol TE/g extract	0.200 mg GAE/g DW	
	*Flammulina velutipes*		-	221 µmol TE/g extract	0.283 mg GAE/g DW	
	*Lentinula edodes*	100 mL (1:20 *w*/*v*), 25 °C, 6 h	-	-	5.06 mg GAE/g DE	[35]
	*Lentinula edodes*	1 mL (80% *v*/*v*), 80 °C, 30 min	-	0.28 µmol/g DW	-	[33]
	*Pleurotus* *ostreatus*	100 mL (80% *v*/*v*), 8 h	-	-	9.64 mg/g extract	[36]
	*Pleurotus eryngii*		-	-	7.91 mg/g extract	
Conv. (Ethanol)	*Agaricus brasiliensis* *Agaricus campestris* *Agaricus silvaticus* *Agaricus bisporus*	Ethanol 70%, 3 h shaking, room temperature	-	-	1154.7 mg GAE/100 g DW 767.3 mg GAE/100 g DW 638.3 mg GAE/100 g DW 132.7 mg GAE/100 g DW	[27]
PEF	*Lentinula edodes* *Pleurotus ostreatus* *Agaricus brunnescens*	200 mL water, specific energy 50 kJ/kg, 2.5 kV/cm + 6 h stirring	228.07 µmol TE/g DW 180.41 µmol TE/g DW 202.18 µmol TE/g DW	62.50 µmol TE/g DW 25.33 µmol TE/g DW 41.01 µmol TE/g DW	24.68 mg GAE/g DW 17.69 µmol TE/g DW 21.68 µmol TE/g DW	Present study
	*Agaricus bisporus*	Specific energy 50 kJ/kg 2.5 and 3 kV/cm (TPC) + 5.6 h in water	161.41 µmol TE/g DW	67.94 µmol TE/g DW	22.16 mg GAE/g DW	[15]
		38.4 kV/cm; 85 °C	-	-	1.6 mg GAE/g mushroom	[26]
MAE	*Terfezia boudieri*	Methanol 80%, 80 °C, 5 min	-	-	182.2 µmol TE/g	[37]
	*Boletus edulis*		-	-	357.7 µmol TE/g	
	*Lactarius volemu*		-	-	230.2 µmol TE/g	
USN	*Boletus edulis*	50% ethanol 45 °C, 40 min	-	-	41.82 mg GAE/g	[28]
	*Boletus auranticus*		-	-	36.43 mg GAE/g	

ORAC: oxygen radical absorbance capacity. TEAC: Trolox equivalent antioxidant capacity. TPC: total phenolic compounds. PEF: pulsed electric fields. MAE: microwave-assisted extraction. USN: ultrasounds. GAE: gallic acid equivalents. TE: Trolox equivalents. DE: dry extract. DW: dry weight.

### 3.2. Phenolic Profile

**Figure 2:**
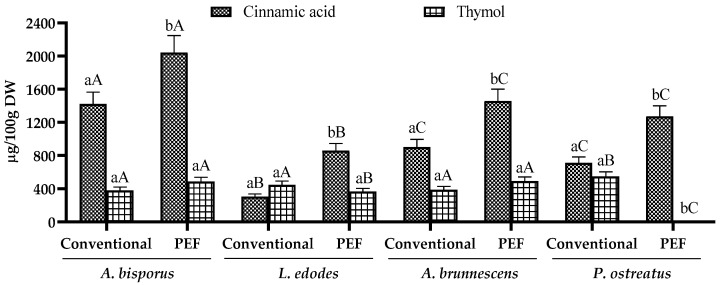
Cinnamic acid (µg/100 g DW) and thymol (µg/100 g DW) content in conventional and pulsed electric field-assisted extraction (PEF) of *A. bisporus*, *L. edodes*, *A. brunnescens*, and *P. ostreatus*. Different lowercase letters indicate significant differences (*p* < 0.05) between extraction methodologies. Different capital letters indicate significant differences (*p* < 0.05) between mushrooms.

The results of the phenolic profile common to all mushroom extracts obtained by high-performance liquid chromatography coupled with mass spectrometry (HPLC-MS) are shown in Figure 2, as well as their chromatographs in Figures S2 and S3. As can be seen, all mushroom extracts contained cinnamic acid ranging from 305.55 ± 15.23 for the conventional methodology to 2043.26 ± 103.02 µg/100 g DW for PEF-assisted extraction and thymol ranging from 366.64 ± 26.54 to 548.73 ± 37.98 µg/100 g DW, based on PEF-assisted extraction and the conventional method, respectively.

Regarding the mushroom species, *A. bisporus* had the highest cinnamic acid content with 1423.23 ± 126.31 µg/100 g DW in the conventional extract and 2043.26 ± 103.02 µg/100 g DW in the PEF extract, whereas *L. edodes* had the lowest content with 305.55 ± 23.41 and 859.13 ± 76.51 µg/100 g DW, respectively, showing significant differences between the fungi (*p* < 0.05). However, similar low thymol levels were observed in all mushrooms, with the maximum value of 548.73 ± 37.98 µg/100 g DW in *P. ostreatus* showing a significant difference (*p* < 0.05) from the other mushrooms. Thus, different behaviors were observed between cinnamic acid and thymol, with a non-significant difference (*p* > 0.05) for the latter with respect to the other species, except for *P. ostreatus*.

The presence of these compounds in different mushroom species has been reported by several authors. Different species of *Agaricus* (*A. brasiliens*, *A. subrulescens*, and *A. bisporus*) [38,39] and *Pleurotus* (*P. ostreatus*, *P. pulmonarius*, and *P. citrinopileatus*) [40,41,42] showed high contents of coumaric, cinnamic, gallic, caffeic, and ferulic acids, among others, in the fruiting body, which are responsible for the high antioxidant capacities of the extracts associated with beneficial properties with a strong correlation [43]. Among the phenolic compounds, chlorogenic acid/caffeoylquinic acid stands out as one of the main compounds responsible for the antioxidant capacity of mushroom extracts such as *Tricholoma scalpturatum*, *Neolentinus cyathiformis*, *Chlorophyllum agaricoides*, and *Lycoperdon utriforme* [44]. In the study published by Gąsecka et al. [27], different *Agaricus* species showed heterogeneous profiles of identified phenolic acids, highlighting trans-cinnamic acid, caffeic acid, and gallic acid as the dominant ones in *A. silvaticus*, *A. camperstis*, and *A. arvensis*. In addition, the phenolic profile revealed only phenolic acids, among which gallic acid, caffeic acid, and ferulic acid were detected in all species.

**Figure 3:**
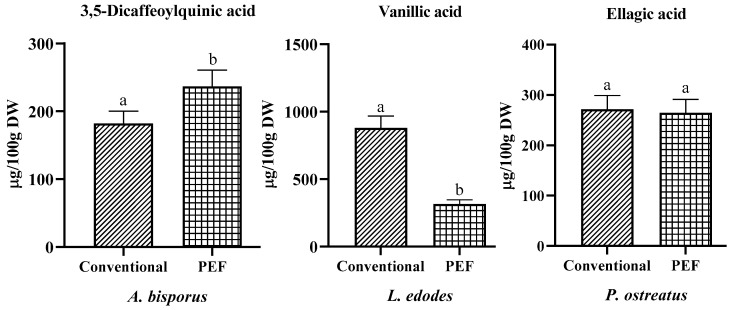
Specific phenolic compounds in conventional and PEF extracts of *A. bisporus*, *L. edodes*, and *P. ostreatus*. Different lowercase letters indicate significant differences (*p* < 0.05) between extraction methods.

In this sense, the specific main compounds found in the aqueous extracts obtained by PEF and conventional extraction from each mushroom are shown in Figure 3. The presence of 3,5-dicaffeoylquinic acid with values of 182.22 ± 20.01 and 236.85 ± 18.45 µg/100 g DW was observed for *A. bisporus*, of vanillic acid with values of 880.32 ± 81.55 and 315.32 ± 28.76 µg/100 g DW for *L. edodes*, and of ellagic acid with values of 271.61 ± 20.45 and 264.66 ± 17.89 µg/100 g DW for *P. ostreatus* obtained by conventional extraction and by PEF, respectively. According to the results, a different influence of PEF on the extraction of specific compounds was observed, where, in the case of 3,5-dicaffeoylquinic acid, a significantly higher yield was obtained by PEF (*p* < 0.05) compared to conventional extraction, whereas the opposite effect was observed for vanillic acid (*p* < 0.05). Moreover, no significant difference was observed between the methods for ellagic acid extraction (*p* > 0.05). These results suggest that the effect of PEF varies depending on the compound of interest, possibly due to different chemical structures and chemical changes during treatment. For this reason, the importance of studying the effects of the conditions and optimizing the extraction for the specific compound of interest, even if the overall antioxidant capacity is increased using PEF since hydroxycinnamic acid derivatives are mostly bound to cell wall components such as lignin, cellulose, and proteins, should be emphasized [45].

### 3.3. Evaluation of Mushroom Surface after PEF Pre-Treatment by Scanning Electron Microscopy

**Figure 4:**
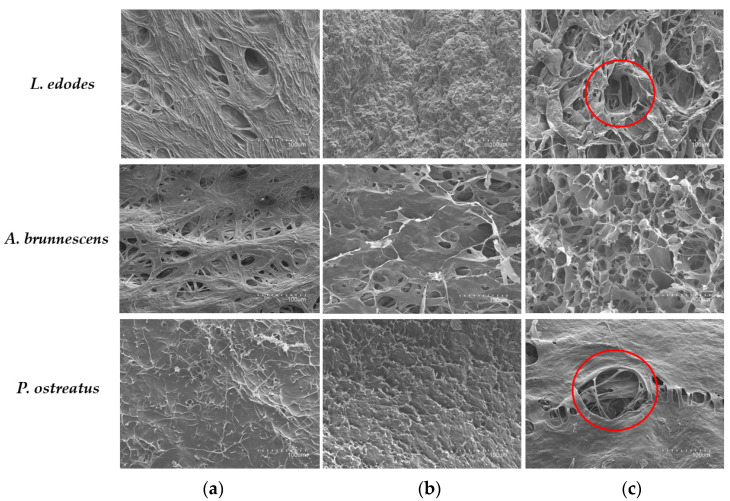
Microstructures of freeze-dried *A. brunnescens, L. edodes*, and *P. ostreatus* after different treatments: (**a**) control sample (no treatment), (**b**) conventional aqueous extraction, and (**c**) pulsed electric field-assisted extraction. All images were obtained by scanning electron microscopy at ×300 magnification.

The surface microstructures of *L. edodes*, *A. brunnescens*, and *P. ostreatus* after PEF-assisted extraction under optimal conditions (50 kJ/kg, 2.5 kV/cm, 6 h) and conventional aqueous extraction (20 °C, 6 h) compared to the control (freeze-dried mushroom) rea shown in Figure 4. As can be seen, the surface of the same mushroom differs depending on the method used and compared to the untreated surface. The surface of the pileus of the control samples presented a fibrous and compact structure, especially in the case of *P. ostreatus*, which could hinder the release of the bioactive components to the external extraction medium.

However, a loss of the fibrous structure was observed during conventional aqueous extraction for 6 h, showing a disorganized aspect that was particularly noticeable in the case of *L. edodes*, whereas the compact structure of *P. ostreatus* was maintained, suggesting that conventional agitation treatment would not be sufficient to affect the surface and largely recover the compounds of interest. On the other hand, PEF technology can cause the surface microporation seen in Figure 4c, which induces a structural change with the presence of cavities that enhances the diffusion of bioactive compounds selectively to the external environment through the disintegration of cell plasma membranes. In this case, the PEF conditions applied were sufficient to produce pores on the surface of *P. ostreatus*, the mushroom with the most compressed surface of the fungi studied.

In this sense, the structural changes induced by the application of PEF and conventional extraction were related to the antioxidant capacity results. As can be seen in Figure 1, when comparing the different mushrooms under the same conditions, the conventional aqueous extraction of *P. ostreatus* showed the lowest recovery of the antioxidant compounds, which could be correlated with the compact structure observed by SEM, suggesting that the conventional method was not sufficient to release the bioactive compounds to the extraction medium. On the other hand, the microporation produced by PEF technology allowed a higher recovery of antioxidant compounds in all samples, highlighting *P. ostreatus*, whose micropores allowed an increase of up to 77.11% for TPC in the selective recovery of these compounds compared to the conventional method despite the compact surface structure.

These results are consistent with those observed by Li et al. [46] and Calleja-Gómez et al. [15] in *L. edodes* and *A. bisporus*, respectively. They suggest that PEF pretreatment significantly alters cell structure through the formation of voids and micropores in the cell wall, which facilitates the release of constituents to the external environment and drying/rehydration of the mushrooms through increased mass transfer. In other matrices, such as Spirulina, pretreatment with PEF also resulted in the formation of pores in the cell wall and breakage of filaments, leading to increased recovery of biomolecules [19].

## 4. Conclusions

From the results obtained, PEF extracts showed a high antioxidant capacity with a strong correlation with phenolic content. In this sense, mushrooms contain mainly phenolic acids such as cinnamic, ellagic, and dicaffeoylquinic acids and monoterpene compounds such as thymol. Therefore, this technique is of interest due to its industrial scalability, being efficient and achieving a greater preservation of these compounds compared to thermal and conventional processes.

In addition, the fibrous microstructure of the fungal pileus was changed by PEF pretreatment, with micropore formation being responsible for the release of substances to the external environment. This is different from the conventional aqueous method, which causes disorganization of the structure.

However, despite the overall effectiveness of the method, the behavior of specific compounds differs in terms of their structure and possible interactions with other cellular components. For this reason, the target molecule should be further studied to determine the optimal conditions of the technology, the optimal matrix for its recovery, and the stability of the extracted polyphenols.

## Data Availability

The data presented in this study is partially contained within the article and Supplementary Material and is available on request from the corresponding author due to privacy.

## Supplementary Materials

The following supporting information can be downloaded at: https://www.mdpi.com/article/10.3390/antiox12122080/s1, Table S1: Phenolic compounds quantified and identified in pulsed electric fields (PEF) extracts and conventional extracts from *Agaricus bisporus, Agaricus brunnescens, Lentinula edodes* and *Pleurotus ostreatus* mushrooms by Triple TOF-LC-MS-MS; Figure S1: LC- MS/MS IDA Survey from (**A**) *A. brunnescens* conventional extract, (**B**) *A. brunnescens* PEF extract, (**C**) *P. ostreatus* conventional extract, (**D**) *P. ostreatus* PEF extract, (**E**) *A. bisporus* conventional extract, (**F**) *A. bisporus* PEF extract, (**G**) *L. edodes* conventional extract, (**H**) *L. edodes* PEF extract; Figure S2: LC- MS/MS cinnamic acid chromatographs from (**A**) *A. brunnescens* conventional extract, (**B**) *A. brunnescens* PEF extract, (**C**) *P. ostreatus* conventional extract, (**D**) *P. ostreatus* PEF extract, (**E**) *A. bisporus* conventional extract, (**F**) *A. bisporus* PEF extract, (**G**) *L. edodes* conventional extract, (**H**) *L. edodes* PEF extract.; Figure S3: LC- MS/MS thymol chromatographs from (**A**) *A. brunnescens* conventional extract, (**B**) *A. brunnescens* PEF extract, (**C**) *P. ostreatus* conventional extract, (**D**) *A. bisporus* conventional extract, (**E**) *A. bisporus* PEF extract, (**F**) *L. edodes* conventional extract, (**G**) *L. edodes* PEF extract; Figure S4: LC- MS/MS standard calibration of (**A**) Kaempferol and (**B**) Hesperidin with sample data showed below.

## References

[1] Menon R. (2014). Oxidative stress damage as a detrimental factor in preterm birth pathology. Front. Immunol..

[2] Betteridge D.J. (2000). What is oxidative stress?. Metabolism.

[3] Mazzocchi A., De Cosmi V., Risé P., Milani G.P., Turolo S., Syrén M.L., Sala A., Agostoni C. (2021). bioactive compounds in edible oils and their role in oxidative stress and inflammation. Front. Physiol..

[4] Liang B., Zhu Y.C., Lu J., Gu N. (2021). Effects of traditional chinese medication-based bioactive compounds on cellular and molecular mechanisms of oxidative stress. Oxid. Med. Cell. Longev..

[5] Royse D.J., Baars J., Tan Q. (2017). Current overview of mushroom production in the world. Edible and Medicinal Mushrooms: Technology and Applications.

[6] Singh M., Kamal S., Sharma V.P. (2021). Status and trends in world mushroom production-III -World production of different mushroom species in 21st century. Mushroom Res..

[7] De Cianni R., Pippinato L., Mancuso T. (2023). A Systematic review on drivers influencing consumption of edible mushrooms and innovative mushroom containing products. Appetite.

[8] Interreg Europe Mushroom Sector as Part of Circular Economy. https://www.interregeurope.eu/good-practices/mushroom-sector-as-part-of-circular-economy.

[9] Reis F.S., Martins A., Barros L., Ferreira I.C.F.R. (2012). Antioxidant properties and phenolic profile of the most widely appreciated cultivated mushrooms: A comparative study between in vivo and in vitro samples. Food Chem. Toxicol..

[10] Mujić I., Zeković Z., Lepojević Ž., Vidović S., Živković J. (2010). Antioxidant properties of selected edible mushroom species. JCEA.

[11] Ferreira I.C.F.R., Barros L., Abreu R.M.V. (2009). Antioxidants in wild mushrooms. Curr. Med. Chem..

[12] Jayakumar T., Thomas P.A., Sheu J.R., Geraldine P. (2011). In-vitro and in-vivo antioxidant effects of the oyster mushroom Pleurotus ostreatus. Food Res. Int..

[13] Gil-Ramírez A., Pavo-Caballero C., Baeza E., Baenas N., Garcia-Viguera C., Marín F.R., Soler-Rivas C. (2016). Mushrooms do not contain flavonoids. J. Funct. Foods.

[14] Roselló-Soto E., Parniakov O., Deng Q., Patras A., Koubaa M., Grimi N., Boussetta N., Tiwari B.K., Vorobiev E., Lebovka N. (2016). Application of non-conventional extraction methods: Toward a sustainable and green production of valuable compounds from mushrooms. Food Eng. Rev..

[15] Calleja-Gómez M., Castagnini J.M., Carbó E., Ferrer E., Berrada H., Barba F.J. (2022). Evaluation of pulsed electric field-assisted extraction on the microstructure and recovery of nutrients and bioactive compounds from mushroom (*Agaricus bisporus*). Separations.

[16] Parniakov O., Lebovka N.I., Van Hecke E., Vorobiev E. (2014). Pulsed electric field assisted pressure extraction and solvent extraction from mushroom (*Agaricus bisporus*). Food Bioproc. Tech..

[17] Nowacka M., Tappi S., Wiktor A., Rybak K., Miszczykowska A., Czyzewski J., Drozdzal K., Witrowa-Rajchert D., Tylewicz U. (2019). The impact of pulsed electric field on the extraction of bioactive compounds from beetroot. Foods.

[18] Usman I., Hussain M., Imran A., Afzaal M., Saeed F., Javed M., Afzal A., Ashfaq I., Al Jbawi E.A., Saewan S. (2022). Traditional and innovative approaches for the extraction of bioactive compounds. Int. J. Food Prop..

[19] Wang M., Zhou J., Collado M.C., Barba F.J. (2021). Accelerated solvent extraction and pulsed electric fields for valorization of rainbow trout (*Oncorhynchus mykiss*) and sole (*Dover sole*) by-products: Protein content, molecular weight distribution and antioxidant potential of the extracts. Mar. Drugs.

[20] Miller N.J., Rice-Evans C., Davies M.J., Gopinathan V., Milner A. (1993). A novel method for measuring antioxidant capacity and its application to monitoring the antioxidant status in premature neonates. Clin. Sci..

[21] Cao G., Alessio H.M., Cutler R.G. (1993). Oxygen-radical absorbance capacity assay for antioxidants. Free Radic. Biol. Med..

[22] Singleton V.L., Orthofer R., Lamuela-Raventós R.M. (1999). Analysis of total phenols and other oxidation substrates and antioxidants by means of Folin-Ciocalteu reagent. Methods Enzymol..

[23] Roselló-Soto E., Martí-Quijal F.J., Cilla A., Munekata P.E.S., Lorenzo J.M., Remize F., Barba F.J. (2019). Influence of temperature, solvent and pH on the selective extraction of phenolic compounds from tiger nuts by-products: Triple-TOF-LC-MS-MS characterization. Molecules.

[24] Redondo D., Venturini M.E., Luengo E., Raso J., Arias E. (2018). Pulsed electric fields as a green technology for the extraction of bioactive compounds from thinned peach by-products. Innov. Food Sci. Emerg. Technol..

[25] Martí-Quijal F.J., Castagnini J.M., Ruiz M.J., Barba F.J. (2023). Sea Bass Side Streams Extracts Obtained by Pulsed Electric Fields: Nutritional Characterization and Effect on SH-SY5Y Cells. Foods.

[26] Xue D., Farid M.M. (2015). Pulsed electric field extraction of valuable compounds from white button mushroom (*Agaricus bisporus*). Innov. Food Sci. Emerg. Technol..

[27] Gąsecka M., Magdziak Z., Siwulski M., Mleczek M. (2018). Profile of phenolic and organic acids, antioxidant properties and ergosterol content in cultivated and wild growing species of Agaricus. Eur. Food Res. Technol..

[28] Vidović S.S., Mujić I.O., Zeković Z.P., Lepojević Ž.D., Tumbas V.T., Mujić A.I. (2010). Antioxidant properties of selected Boletus mushrooms. Food Biophys..

[29] Barba F.J., Galanakis C.M., Esteve M.J., Frigola A., Vorobiev E. (2015). Potential use of pulsed electric technologies and ultrasounds to improve the recovery of highadded value compounds from blackberries. J. Food Eng..

[30] Anticona M., Blesa J., Frigola A., Esteve M.J. (2020). High biological value compounds extraction from citrus waste with non-conventional methods. Foods.

[31] Barba F.J., Zhu Z., Koubaa M., Sant’Ana A.S., Orlien V. (2016). Green alternative methods for the extraction of antioxidant bioactive compounds from winery wastes and by-products: A review. Trends Food Sci. Technol..

[32] Shen L., Pang S., Zhong M., Sun Y., Qayum A., Liu Y., Rashid A., Xu B., Liang Q., Ma H. (2023). A Comprehensive Review of Ultrasonic Assisted Extraction (UAE) for Bioactive Components: Principles, Advantages, Equipment, and Combined Technologies. Ultrason Sonochem..

[33] Wu X.J., Hansen C. (2008). Antioxidant capacity, phenolic content, and polysaccharide content of *Lentinus edodes* grown in whey permeate-based submerged culture. J. Food Sci..

[34] Zeng X., Suwandi J., Fuller J., Doronila A., Ng K. (2012). Antioxidant capacity and mineral contents of edible wild Australian mushrooms. Food Sci. Technol. Int..

[35] Nam M., Choi J.Y., Kim M.S. (2021). Metabolic profiles, bioactive compounds, and antioxidant capacity in *Lentinula edodes* cultivated on log versus sawdust substrates. Biomolecules.

[36] Gąsecka M., Mleczek M., Siwulski M., Niedzielski P. (2016). Phenolic composition and antioxidant properties of *Pleurotus ostreatus* and *Pleurotus eryngii* enriched with selenium and zinc. Eur. Food Res. Technol..

[37] Özyürek M., Bener M., Güçlü K., Apak R. (2014). Antioxidant/antiradical properties of microwave-assisted extracts of three wild edible mushrooms. Food Chem..

[38] Bach F., Zielinski A.A.F., Helm C.V., Maciel G.M., Pedro A.C., Stafussa A.P., Ávila S., Haminiuk C.W.I. (2019). Bio compounds of edible mushrooms: In vitro antioxidant and antimicrobial activities. LWT.

[39] Sabino Ferrari A.B., Azevedo de Oliveira G., Mannochio Russo H., de Carvalho Bertozo L., da Silva Bolzani V., Cunha Zied D., Farias Ximenes V., Zeraik M.L. (2021). *Pleurotus ostreatus* and *Agaricus subrufescens*: Investigation of chemical composition and antioxidant properties of these mushrooms cultivated with different handmade and commercial supplements. Int. J. Food Sci. Technol..

[40] Gogoi P., Chutia P., Singh P., Mahanta C.L. (2019). Effect of optimized ultrasoundassisted aqueous and ethanolic extraction of *Pleurotus citrinopileatus* mushroom on total phenol, flavonoids and antioxidant properties. J. Food Process Eng..

[41] de Souza Campos Junior F.A., Petrarca M.H., Meinhart A.D., de Jesus Filho M., Godoy H.T. (2019). Multivariate optimization of extraction and validation of phenolic acids in edible mushrooms by capillary electrophoresis. Food Res. Int..

[42] Contato A.G., Inácio F.D., de Araújo C.A.V., Brugnari T., Maciel G.M., Haminiuk C.W.I., Bracht A., Peralta R.M., de Souza C.G.M. (2020). Comparison between the aqueous extracts of mycelium and basidioma of the edible mushroom *Pleurotus pulmonarius*: Chemical composition and antioxidant analysis. J. Food Meas. Charact..

[43] Abdelshafy A.M., Belwal T., Liang Z., Wang L., Li D., Luo Z., Li L. (2021). A comprehensive review on phenolic compounds from edible mushrooms: Occurrence, biological activity, application and future prospective. Crit. Rev. Food Sci. Nutr..

[44] Sezgin S., Dalar A., Uzun Y. (2020). Determination of antioxidant activities and chemical composition of sequential fractions of five edible mushrooms from Turkey. J. Food Sci. Technol..

[45] Çayan F., Deveci E., Tel-Çayan G., Duru M.E. (2020). Identification and quantification of phenolic acid compounds of twenty-six mushrooms by HPLC–DAD. J. Food Meas. Charact..

[46] Li X., Li J., Wang R., Rahaman A., Zeng X.A., Brennan C.S. (2021). Combined effects of pulsed electric field and ultrasound pretreatments on mass transfer and quality of mushrooms. LWT.

