# Supplementary material for: Scanning Electron Microscopy and Triple TOF-LC-MS-MS Analysis of Polyphenols from PEF-Treated Edible Mushrooms (L. edodes, A. brunnescens, and P. ostreatus)
Source: Antioxidants (Basel). 2023 Dec 6;12(12):2080. doi: 10.3390/antiox12122080 (PMC10740608; doi:10.3390/antiox12122080)
Supplement: Supplementary file 1 [file antioxidants-12-02080-s001.zip › antioxidants-2692840-supplementary.pdf]

antioxidants

Supplementary Material

Table S1. Phenolic compounds quantified and identified in pulsed electric fields (PEF) extracts and conventional extracts from *Agaricus bisporus, Agaricus brunnescens, Lentinula edodes* and *Pleurotus ostreatus* mushrooms by Triple TOF-LC-MS-MS.

Compound	Sample	Treatment	Retention time (min)	Concentration (ppm)*
Cinnamic acid	*A. bisporus*	Conventional	1.62	0.254
		PEF	1.64	0.303
	A. brunnescens	Conventional	1.55	0.212
		PEF	1.56	0.256
	L. edodes	Conventional	1.59	0.164
		PEF	1.60	0.209
	P. ostreatus	Conventional	1.55	0.197
		PEF	1.58	0.242
Chlorogenic acid	*A. bisporus*	Conventional	8.39	0.155
		PEF	8.37	0.159
	A. brunnescens	Conventional	n.d	n.d
		PEF	n.d	n.d
	L. edodes	Conventional	n.d	n.d
		PEF	n.d	n.d
	P. ostreatus	Conventional	8.34	0.157
		PEF	n.d	n.d
Vanillic acid	*A. bisporus*	Conventional	n.d	n.d
		PEF	n.d	n.d
	A. brunnescens	Conventional	n.d	n.d
		PEF	n.d	n.d
	L. edodes	Conventional	10.56	0.210
		PEF	10.41	0.165
	P. ostreatus	Conventional	n.d	n.d
		PEF	n.d	n.d
Ellagic acid	*A. bisporus*	Conventional	n.d	n.d
		PEF	n.d	n.d
	A. brunnescens	Conventional	n.d	n.d
		PEF	n.d	n.d
	L. edodes	Conventional	n.d	n.d
		PEF	n.d	n.d

	P. ostreatus	Conventional	8.51	0.162
		PEF	8.41	0.161
Thymol	*A. bisporus*	Conventional	13.52	0.170
		PEF	13.42	0.179
	A. brunnescens	Conventional	13.57	0.171
		PEF	13.66	0.179
	L. edodes	Conventional	13.67	0.176
		PEF	13.33	0.169
	P. ostreatus	Conventional	13.65	0.184
		PEF	n.d	n.d
Hesperidin	*A. bisporus*	Conventional	8.88	7.21E-02
		PEF	8.87	7.24E-02
	A. brunnescens	Conventional	n.d	n.d
		PEF	8.91	7.27E-02
	L. edodes	Conventional	8.91	7.25E-02
		PEF	9.03	7.28E-02
	P. ostreatus	Conventional	n.d	n.d
		PEF	8.91	7.27E-02
Quercetin	*A. bisporus*	Conventional	8.5	3.62E-02
		PEF	8.49	4.53E-02
	A. brunnescens	Conventional	8.4	3.23E-02
		PEF	8.46	4.54E-02
	L. edodes	Conventional	8.49	3.84E-02
		PEF	8.6	3.97E-02
	P. ostreatus	Conventional	8.41	3.13E-02
		PEF	8.51	3.56E-02
Kaempferol	*A. bisporus*	Conventional	10.32	2.31E-03
		PEF	10.31	1.36E-03
	A. brunnescens	Conventional	10.29	4.89E-03
		PEF	11.69	3.14E-03
	L. edodes	Conventional	10.43	6.77E-03
		PEF	10.22	1.70E-02
	P. ostreatus	Conventional	11.68	3.64E-03
		PEF	11.76	2.27E-02

* Phenolic compounds quantification was carried out considering a relative standard deviation (RSD%) of a maximum of 10%.

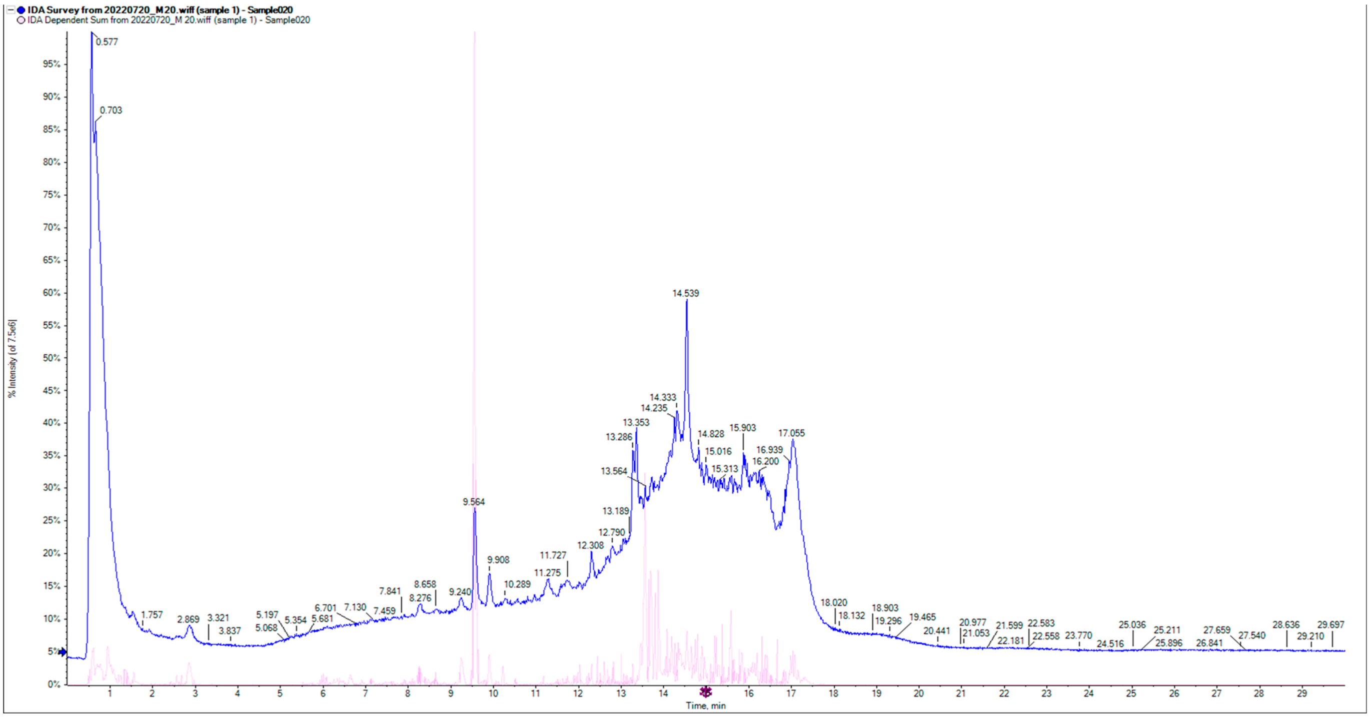
IDA Survey from 20220720_M20.wiff (sample 1) - Sample020
IDA Dependent Sum from 20220720_M 20.wiff (sample 1) - Sample020
% Intensity (of 7.5e6)
Time, min

(A)

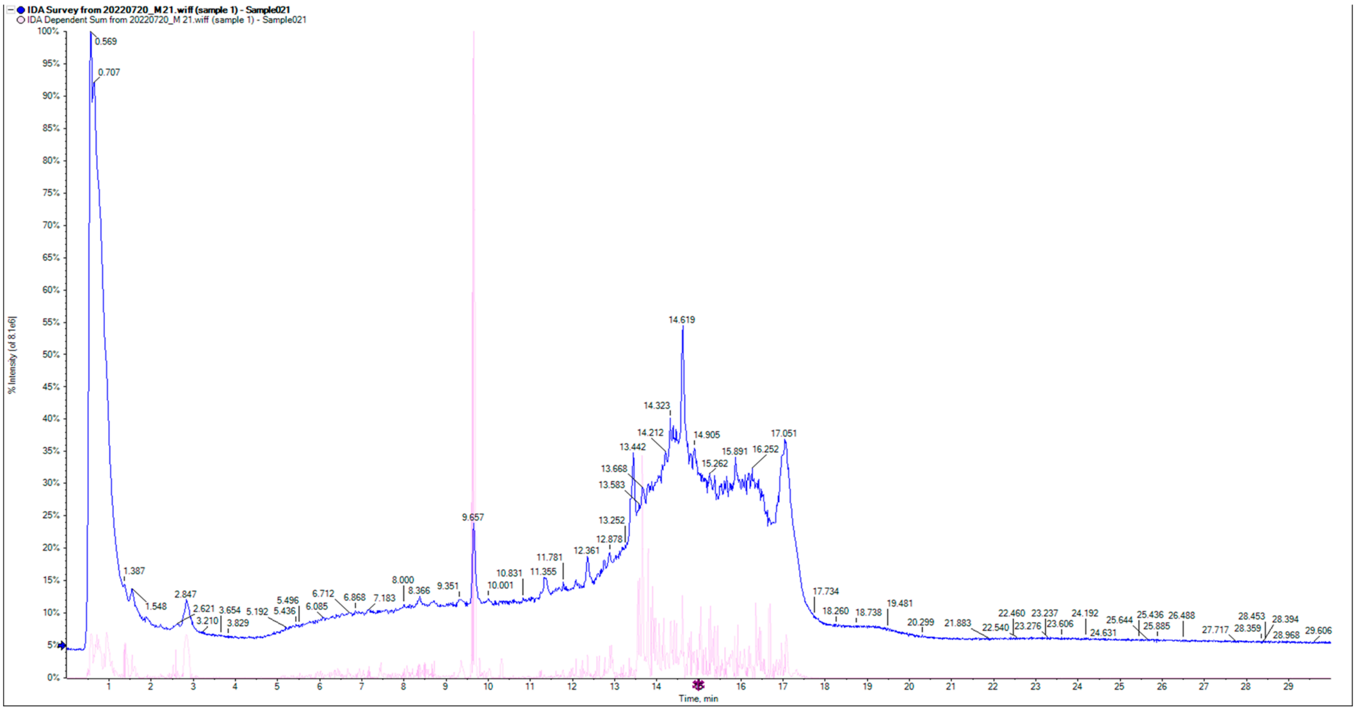
IDA Survey from 20220720_M21.wiff (sample 1) - Sample021
IDA Dependent Sum from 20220720_M 21.wiff (sample 1) - Sample021
% Intensity (of 8.1e6)
Time, min

(B)

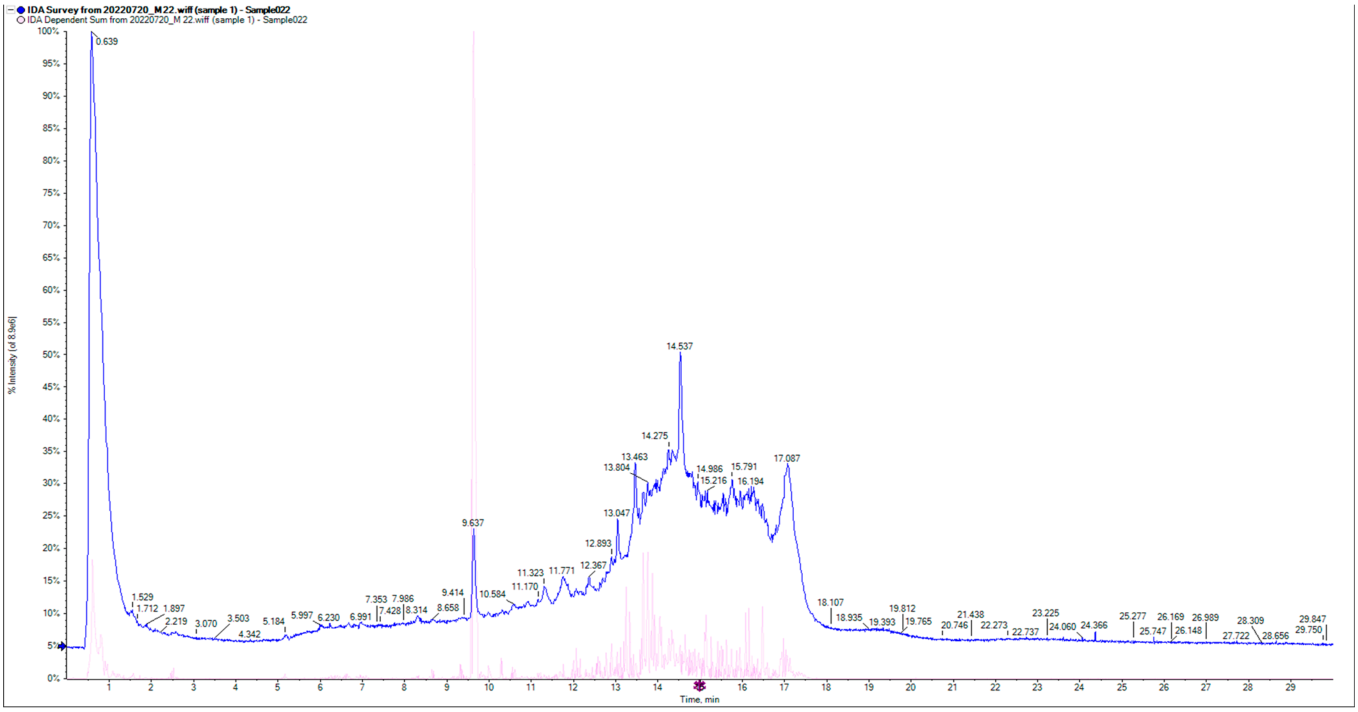
IDA Survey from 20220720_M22.wiff (sample 1) - Sample022
IDA Dependent Sum from 20220720_M 22.wiff (sample 1) - Sample022
% Intensity (of 8.9e6)
Time, min

(C)

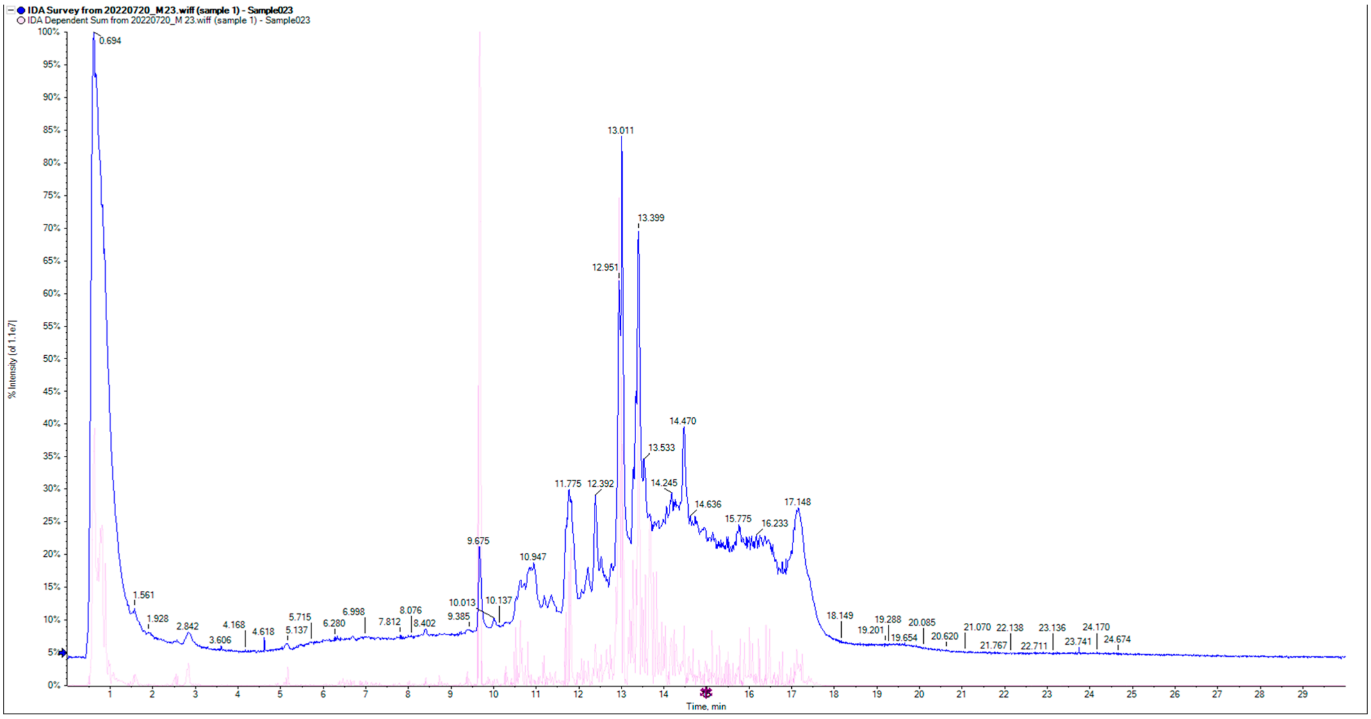
IDA Survey from 20220720_M23.wiff (sample 1) - Sample023
IDA Dependent Sum from 20220720_M 23.wiff (sample 1) - Sample023
% Intensity (of 1.1e7)
Time, min

(D)

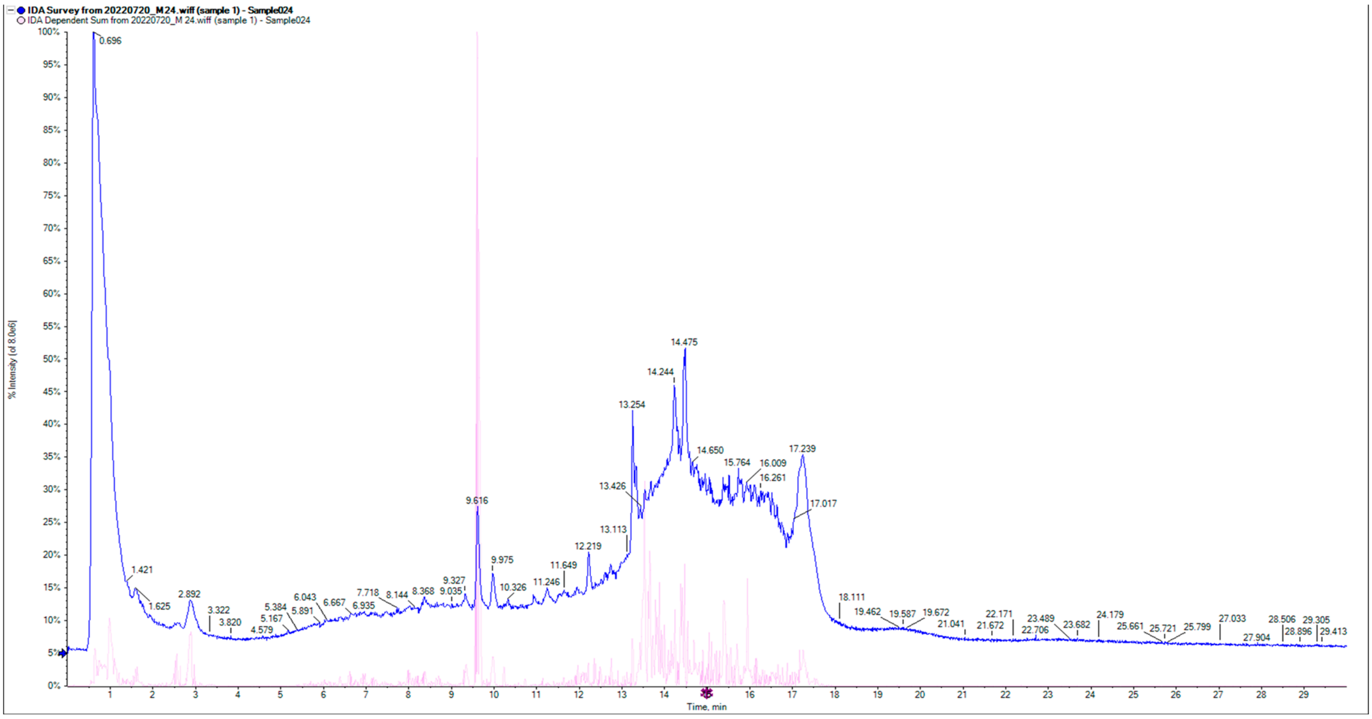
IDA Survey from 20220720_M24.wiff (sample 1) - Sample024
IDA Dependent Sum from 20220720_M24.wiff (sample 1) - Sample024
% Intensity (of 8.0e6)
Time, min

(E)

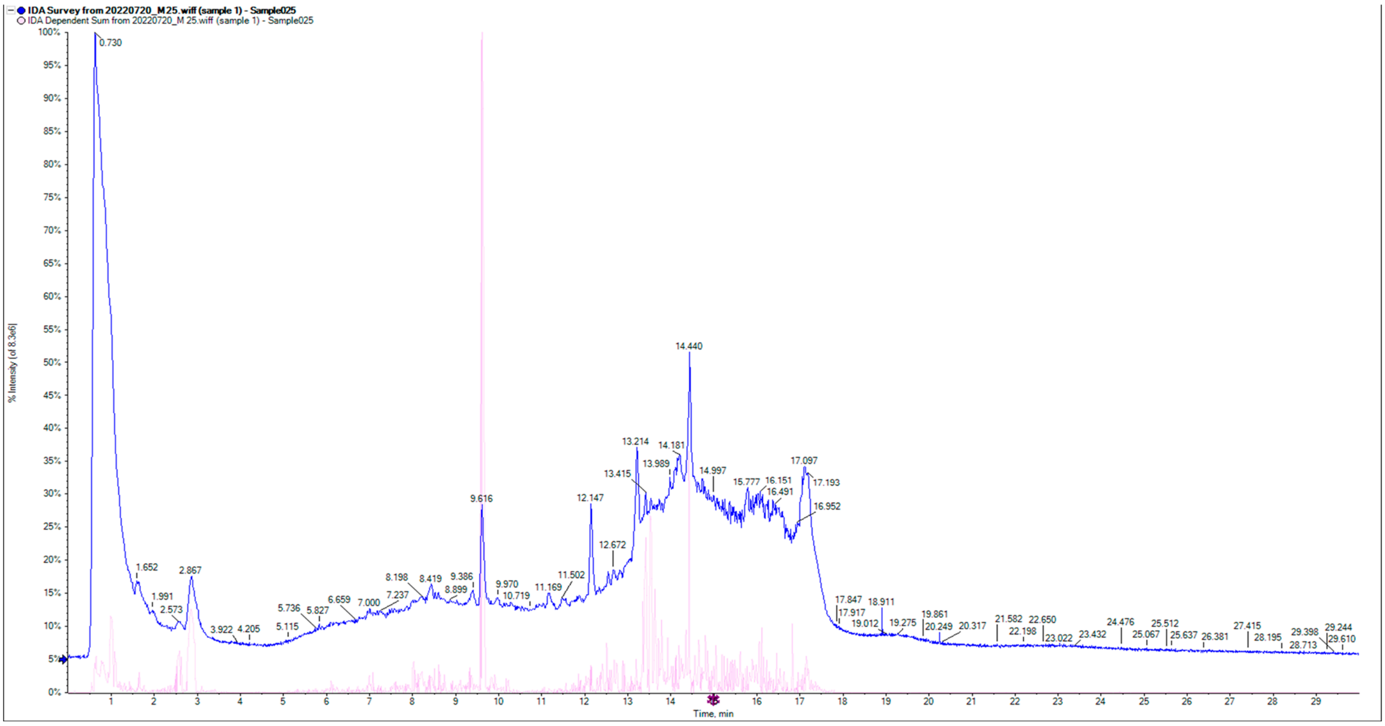
IDA Survey from 20220720_M25.wiff (sample 1) - Sample025
IDA Dependent Sum from 20220720_M25.wiff (sample 1) - Sample025
% Intensity (of 8.3e6)
Time, min

(F)

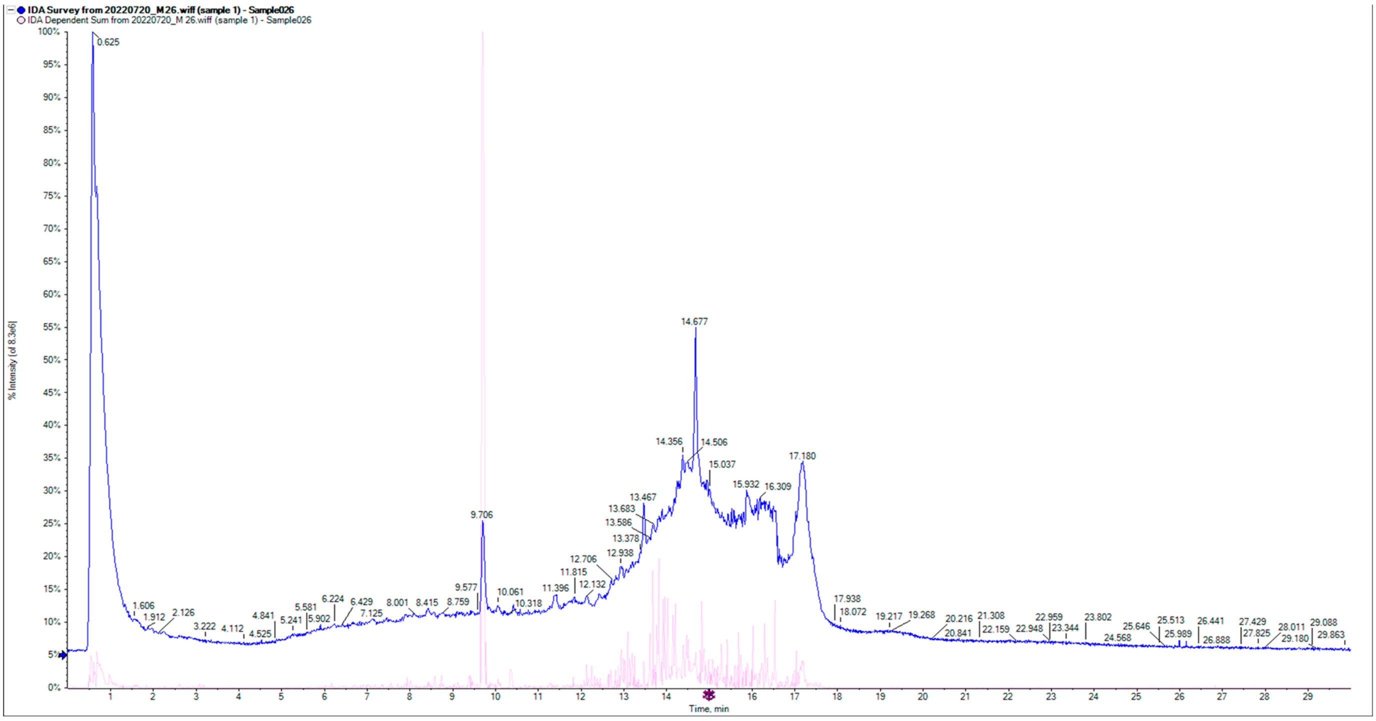

(G)

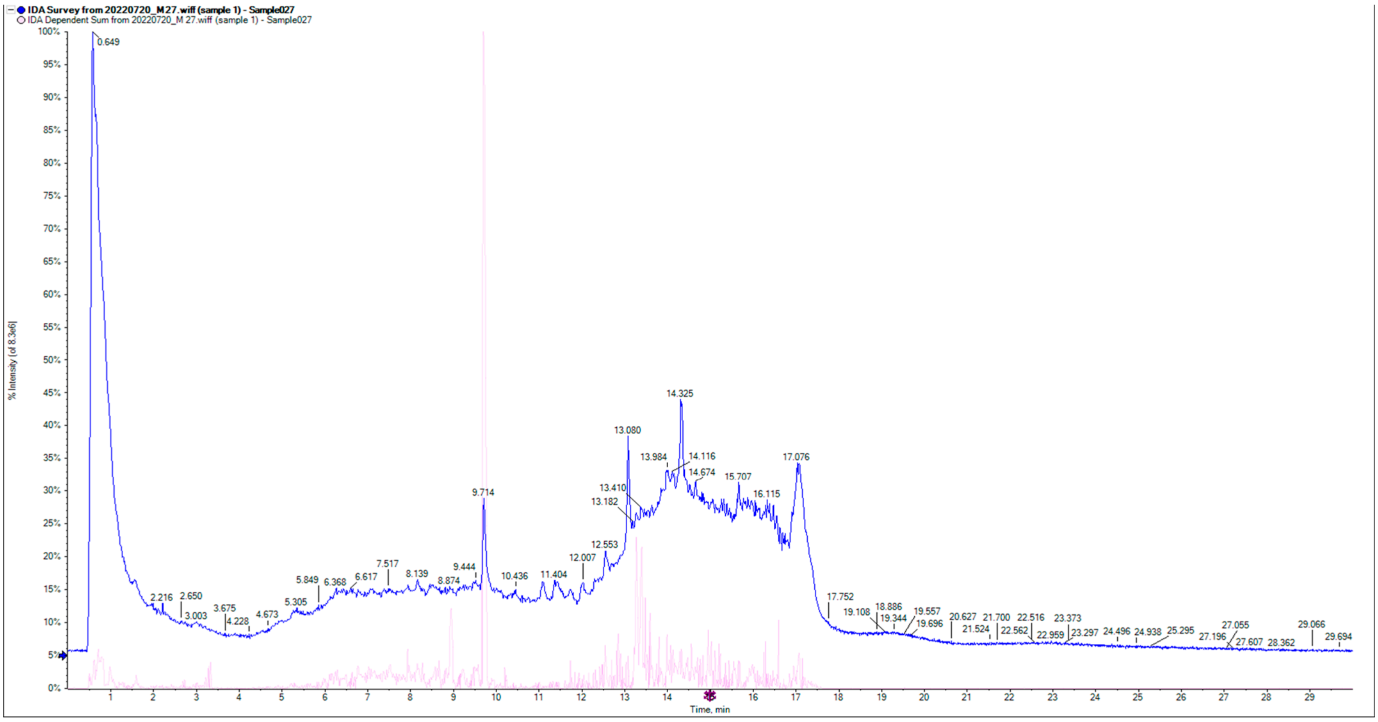

(H)

Figure S1. LC- MS/MS IDA Survey from (**A**) *A. brunnescens* conventional extract, (**B**) *A. brunnescens* PEF extract, (**C**) *P. ostreatus* conventional extract, (**D**) *P. ostreatus* PEF extract, (**E**) *A. bisporus* conventional extract, (**F**) *A. bisporus* PEF extract, (**G**) *L. edodes* conventional extract, (**H**) *L. edodes* PEF extract.

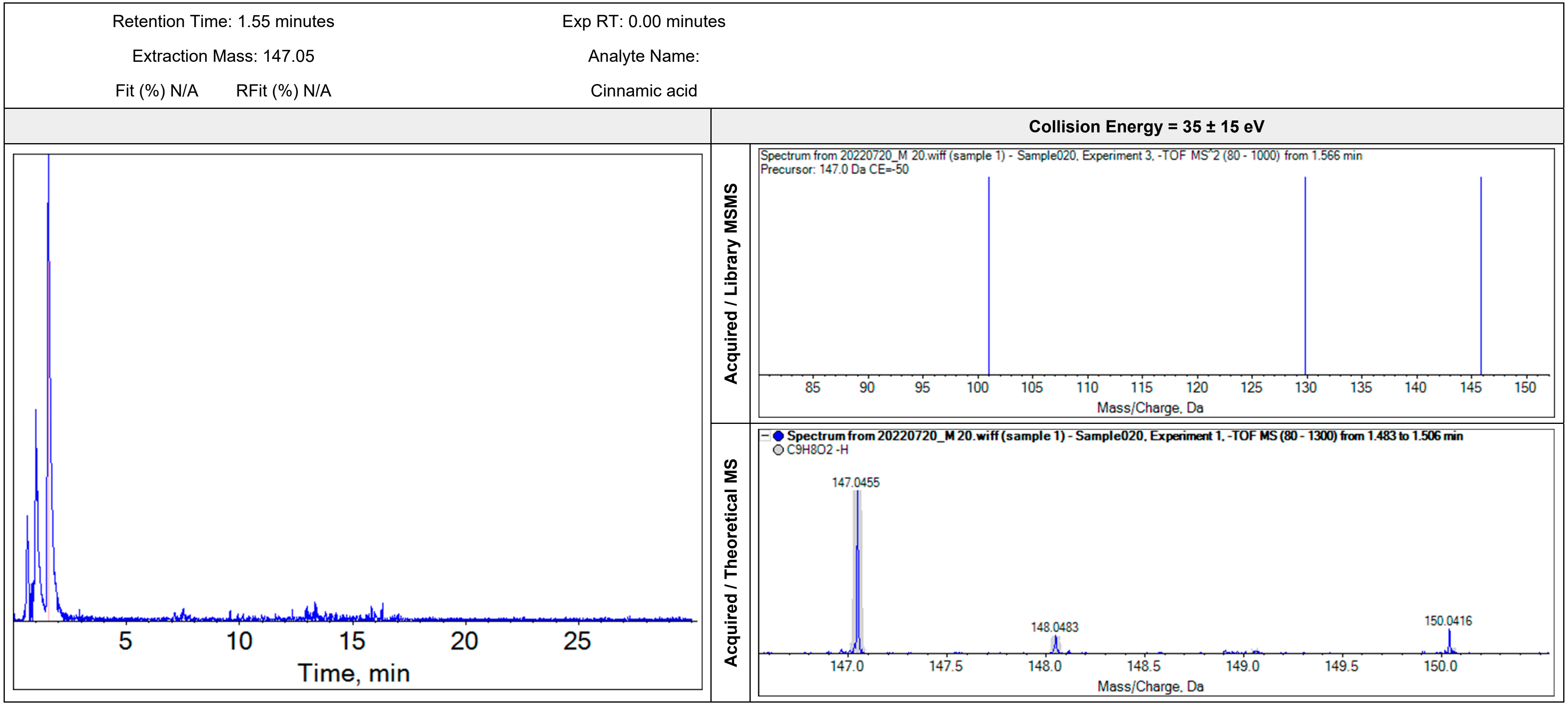
Retention Time: 1.55 minutes
Exp RT: 0.00 minutes
Extraction Mass: 147.05
Analyte Name:
Fit (%) N/A
RFit (%) N/A
Cinnamic acid
Collision Energy = 35 ± 15 eV
Acquired / Library MSMS
Spectrum from 20220720_M 20.wiff (sample 1) - Sample020, Experiment 3, -TOF MS^2 (80 - 1000) from 1.566 min
Precursor: 147.0 Da CE=-50
85
90
95
100
105
110
115
120
125
130
135
140
145
150
Mass/Charge, Da
Acquired / Theoretical MS
Spectrum from 20220720_M 20.wiff (sample 1) - Sample020, Experiment 1, -TOF MS (80 - 1300) from 1.483 to 1.506 min
C9H8O2 -H
147.0455
148.0483
150.0416
147.0
147.5
148.0
148.5
149.0
149.5
150.0
Mass/Charge, Da
5
10
15
20
25
Time, min

(A)

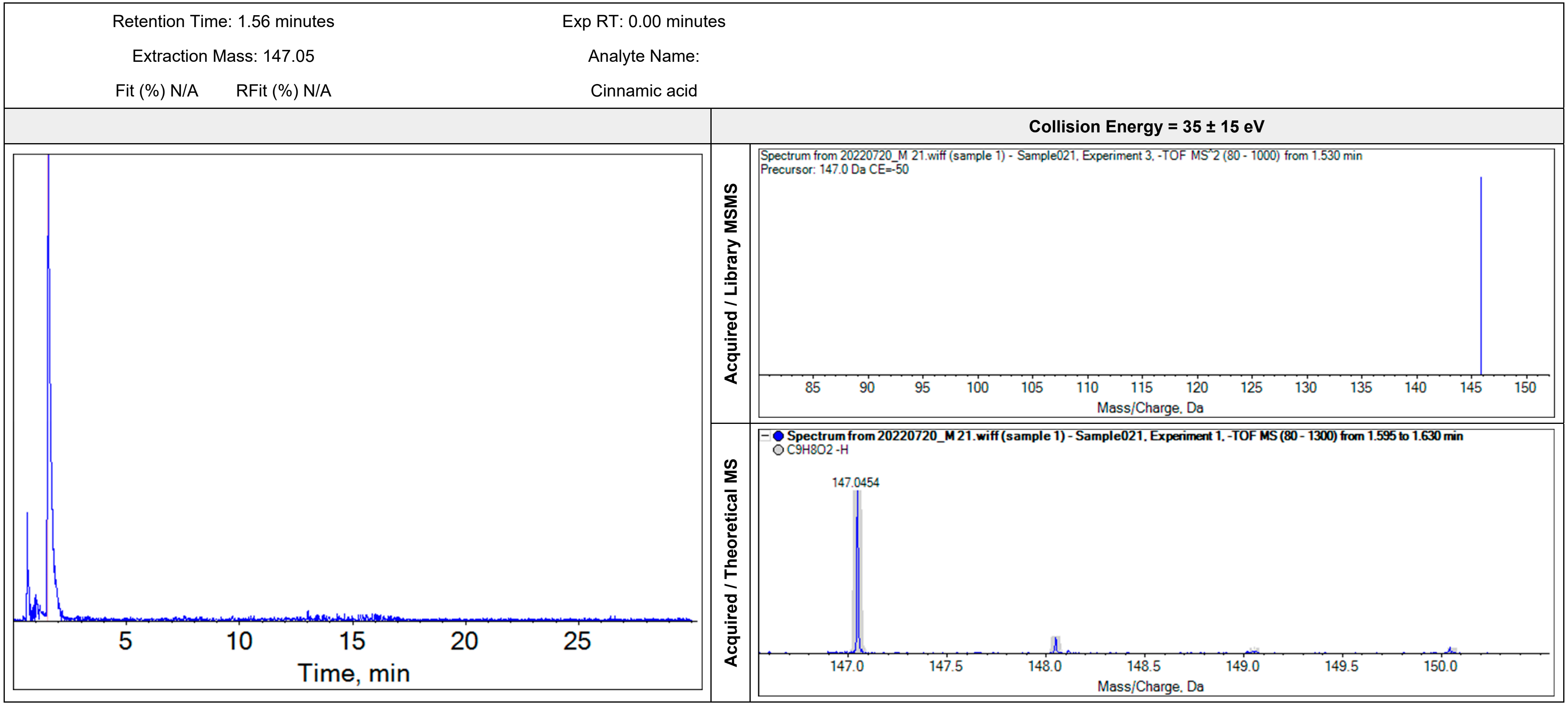

(B)

Retention Time: 1.55 minutes	Exp RT: 0.00 minutes
Extraction Mass: 147.05	Analyte Name:
Fit (%) N/A RFit (%) N/A	Cinnamic acid

		Collision Energy = 35 ± 15 eV
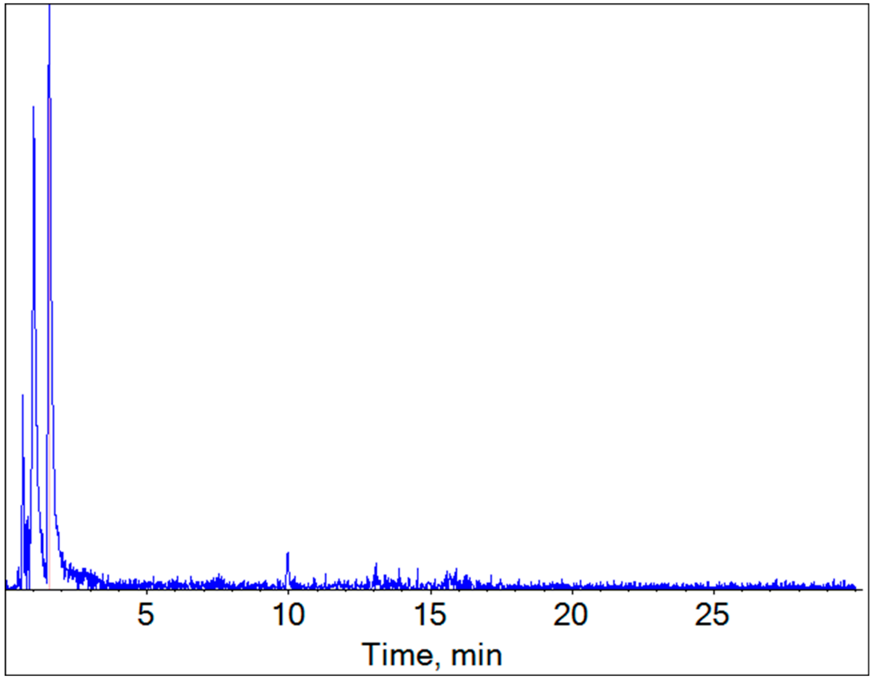	Acquired / Library MSMS	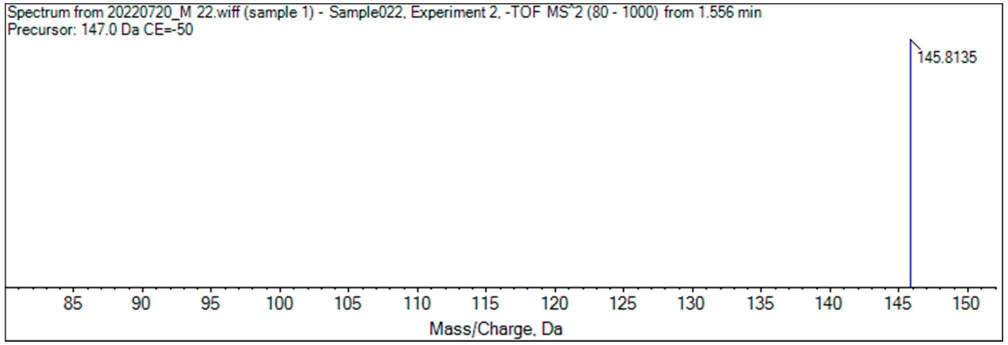
	Acquired / Theoretical MS	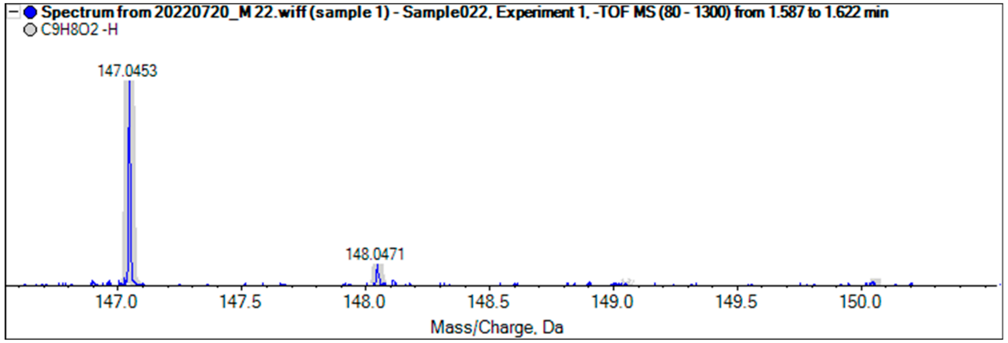

(C)

Retention Time: 1.58 minutes<br>Extraction Mass: 147.05<br>Fit (%) N/A RFit (%) N/A	Exp RT: 0.00 minutes<br>Analyte Name:<br>Cinnamic acid	
	Collision Energy = 35 ± 15 eV	

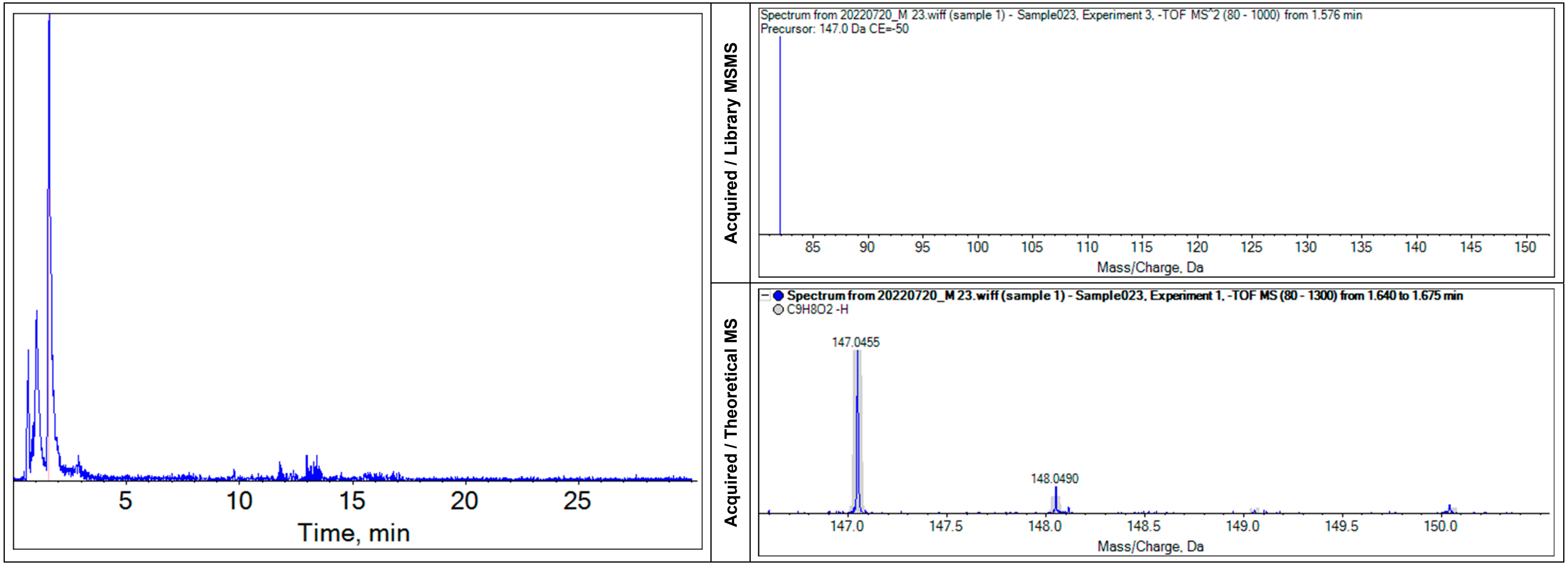

(D)

Retention Time: 1.62 minutes	Exp RT: 0.00 minutes
Extraction Mass: 147.05	Analyte Name:
Fit (%) N/A RFit (%) N/A	Cinnamic acid
	**Collision Energy = 35 ± 15 eV**

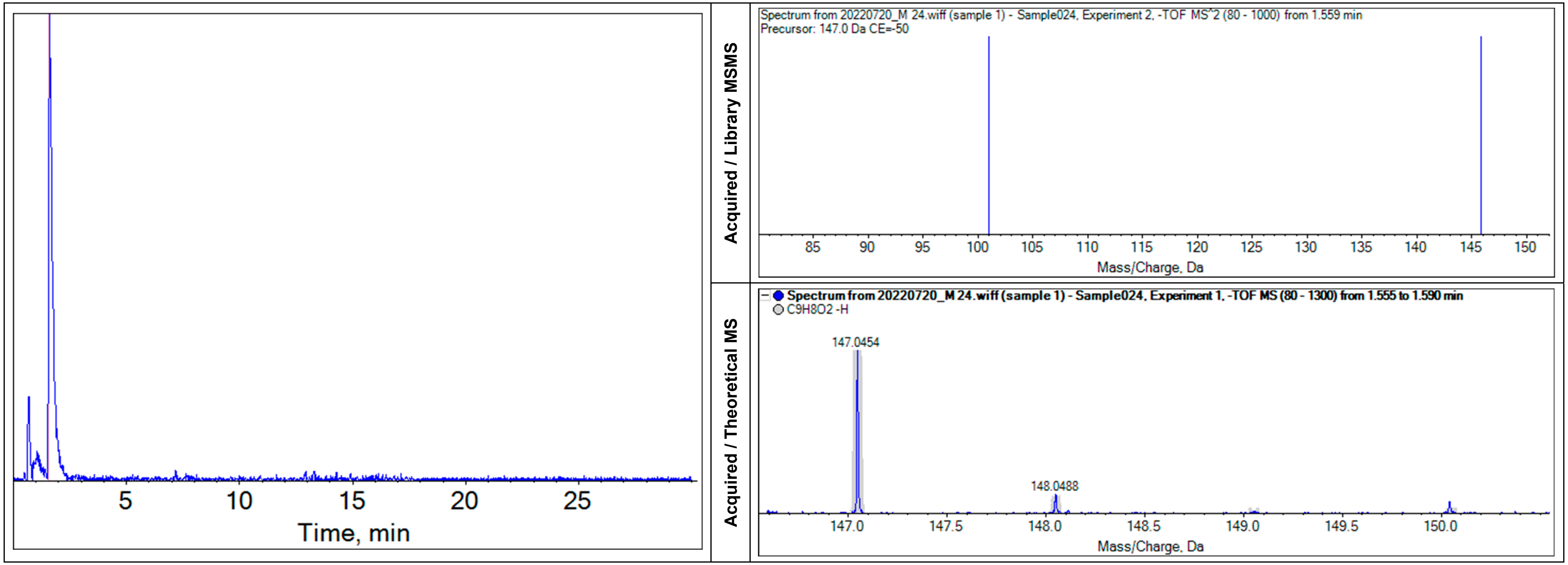

(E)

Retention Time: 1.64 minutes		Exp RT: 0.00 minutes
Extraction Mass: 147.05		Analyte Name:
Fit (%) N/A	RFit (%) N/A	Cinnamic acid
		**Collision Energy = 35 ± 15 eV**

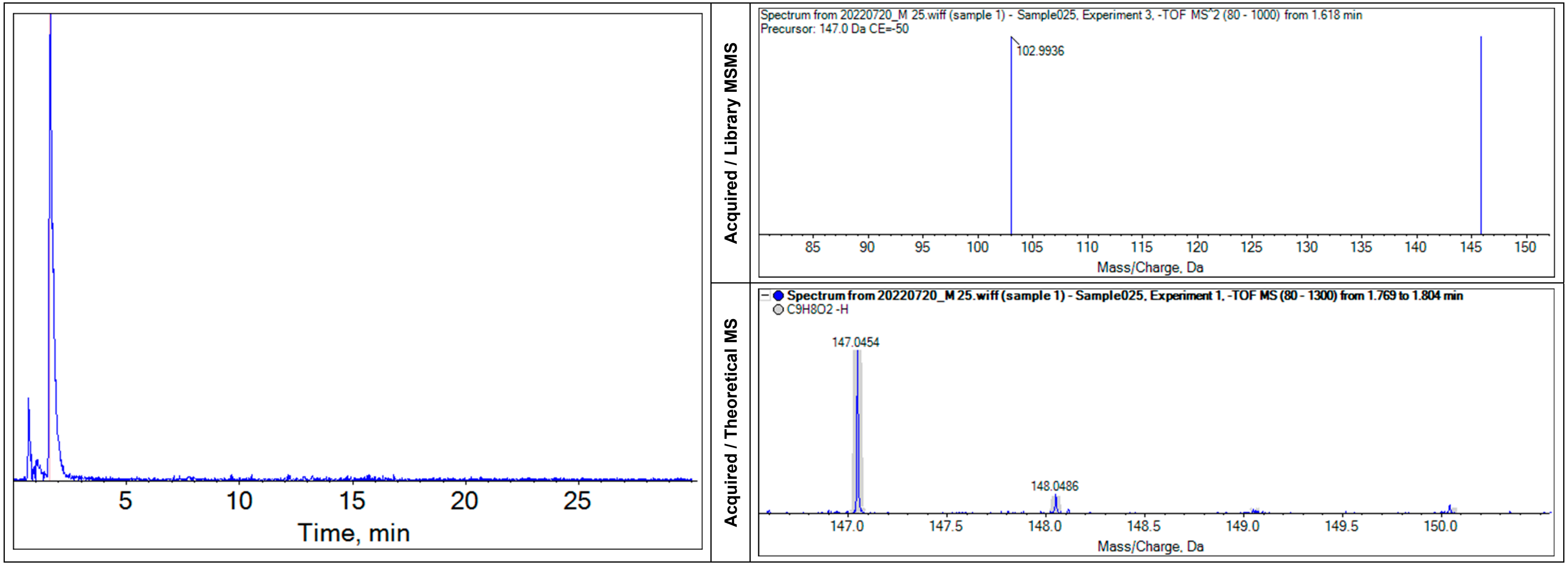

(F)

Retention Time: 1.59 minutes	Exp RT: 0.00 minutes
Extraction Mass: 147.05	Analyte Name:
Fit (%) N/A RFit (%) N/A	Cinnamic acid
	**Collision Energy = 35 ± 15 eV**

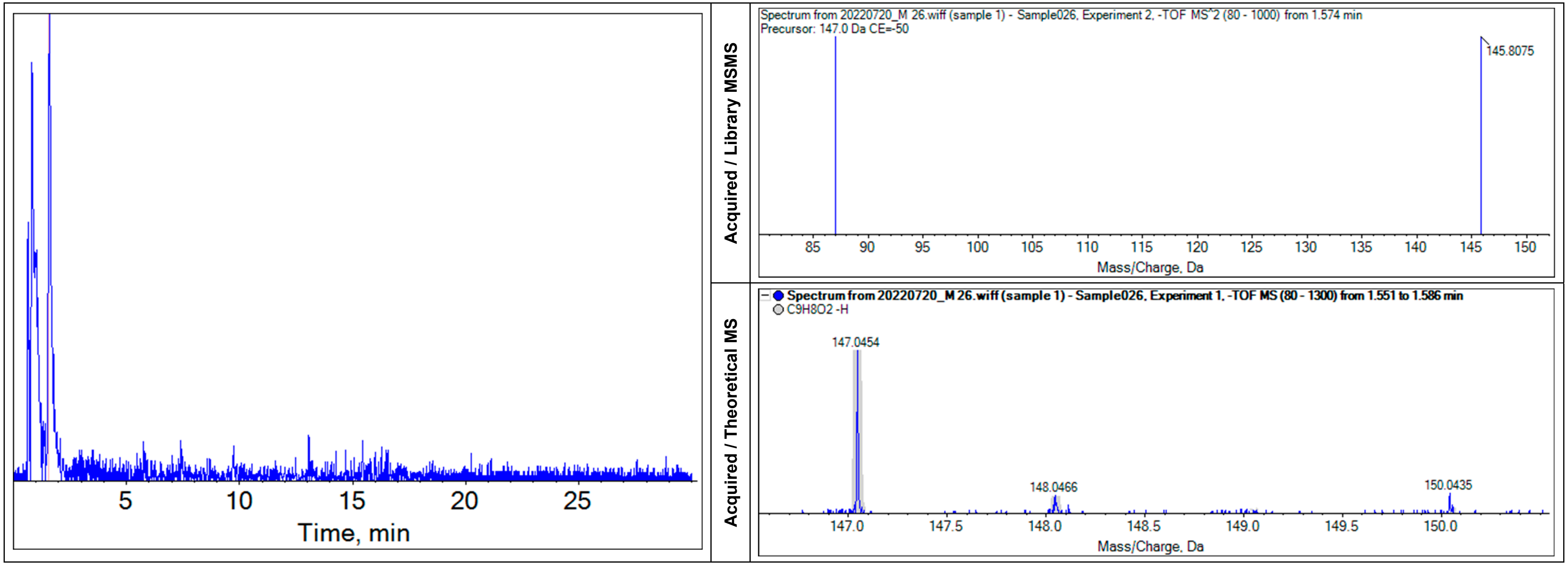

(G)

Retention Time: 1.60 minutes	Exp RT: 0.00 minutes
Extraction Mass: 147.05	Analyte Name:
Fit (%) N/A RFit (%) N/A	Cinnamic acid
	**Collision Energy = 35 ± 15 eV**

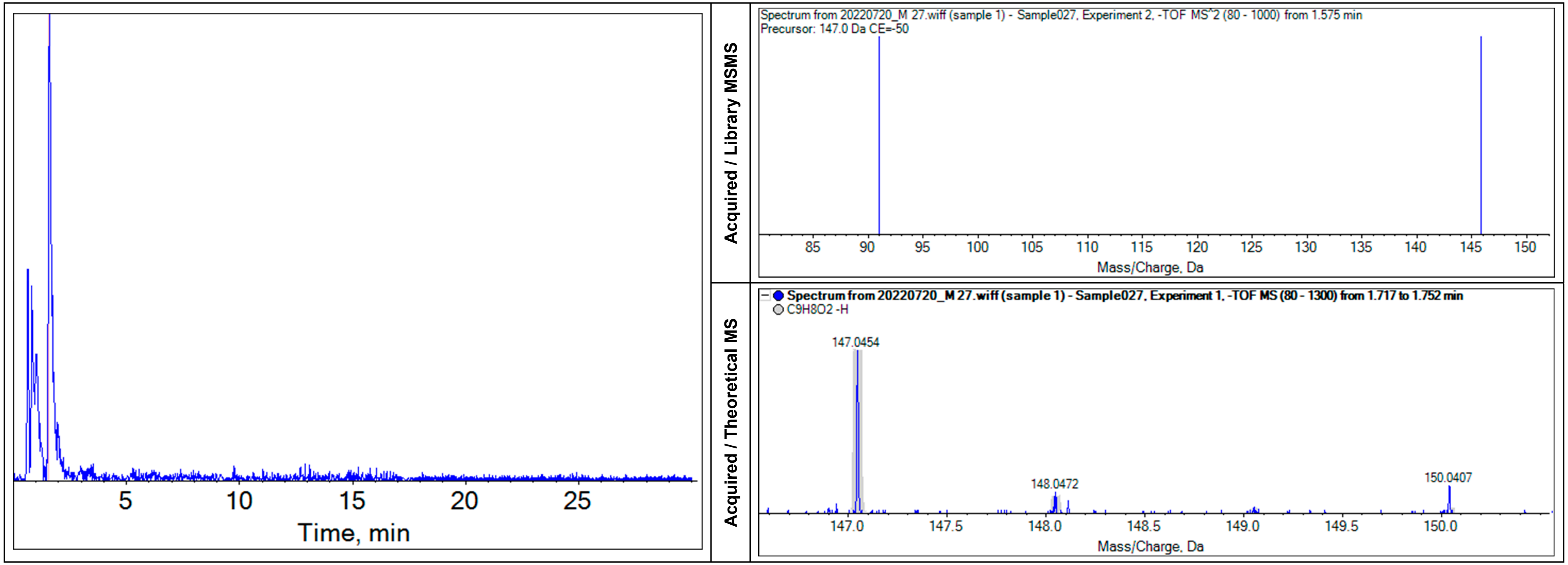

(H)

Figure S2. LC- MS/MS cinnamic acid chromatographs from (**A**) *A. brunnescens* conventional extract, (**B**) *A. brunnescens* PEF extract, (**C**) *P. ostreatus* conventional extract, (**D**) *P. ostreatus* PEF extract, (**E**) *A. bisporus* conventional extract, (**F**) *A. bisporus* PEF extract, (**G**) *L. edodes* conventional extract, (**H**) *L. edodes* PEF extract.

Retention Time: 13.57 minutes	Exp RT: 0.00 minutes
Extraction Mass: 149.10	Analyte Name:
Fit (%) N/A RFit (%) N/A	Thymol
	**Collision Energy = 35 ± 15 eV**

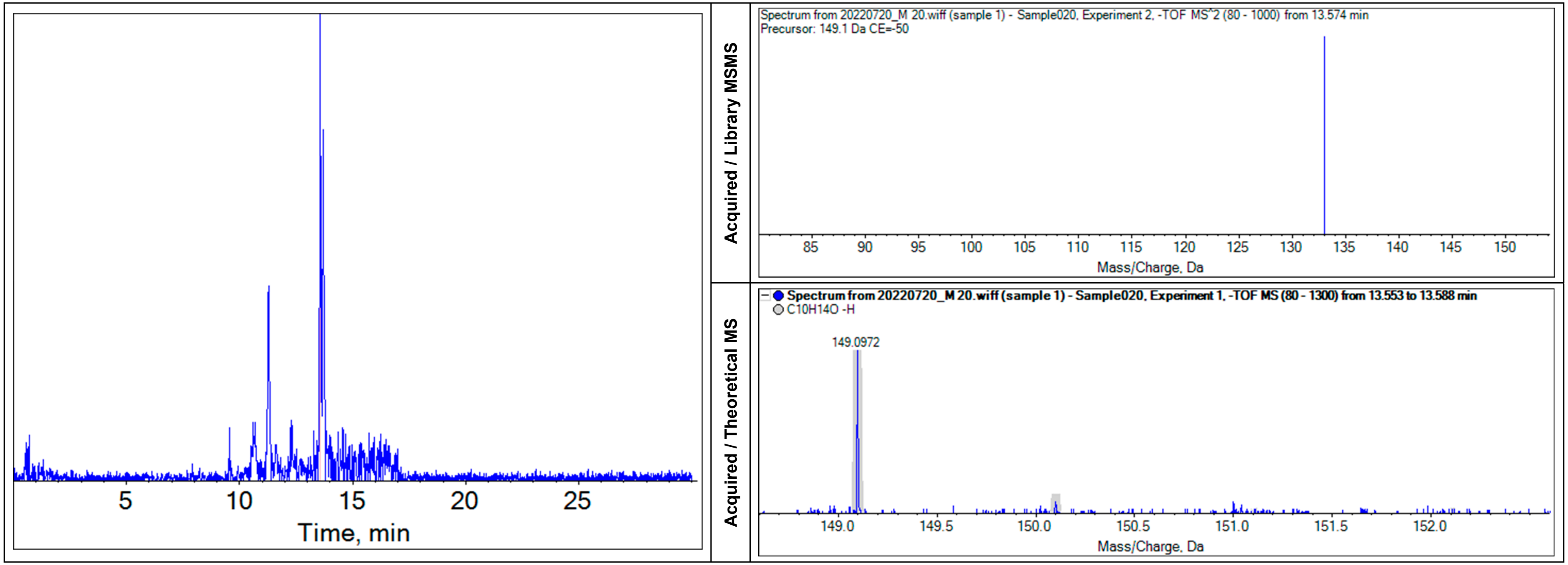

(A)

Retention Time: 13.66 minutes	Exp RT: 0.00 minutes
Extraction Mass: 149.10	Analyte Name:
Fit (%) N/A RFit (%) N/A	Thymol
	**Collision Energy = 35 ± 15 eV**

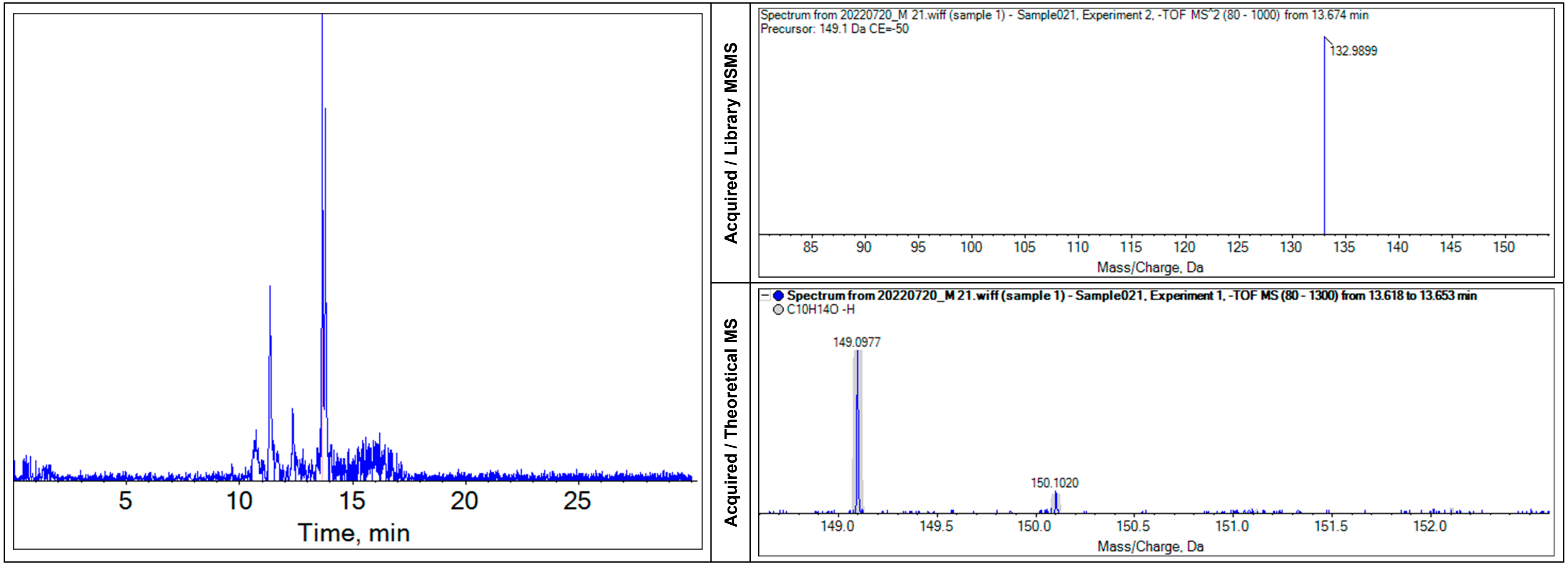

(B)

Retention Time: 13.65 minutes	Exp RT: 0.00 minutes
Extraction Mass: 149.10	Analyte Name:
Fit (%) N/A RFit (%) N/A	Thymol
	**Collision Energy = 35 ± 15 eV**

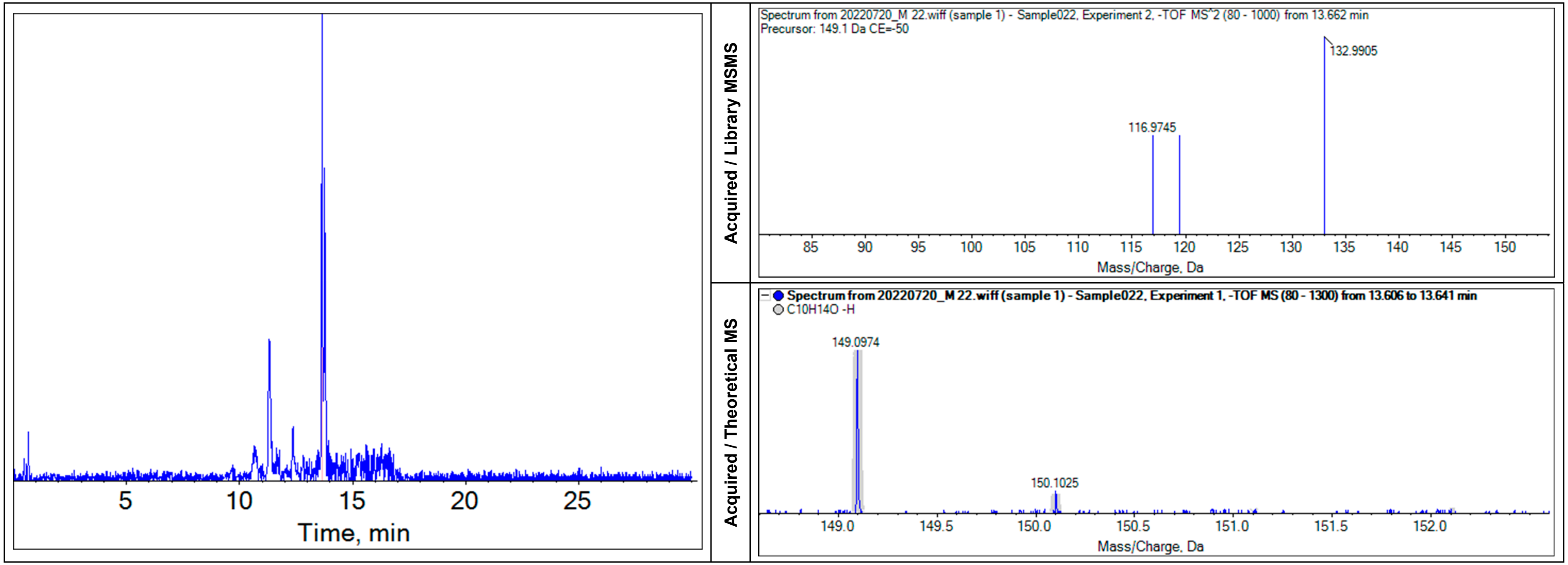

(C)

Retention Time: 13.53 minutes	Exp RT: 0.00 minutes
Extraction Mass: 149.10	Analyte Name:
Fit (%) N/A RFit (%) N/A	Thymol
	**Collision Energy = 35 ± 15 eV**

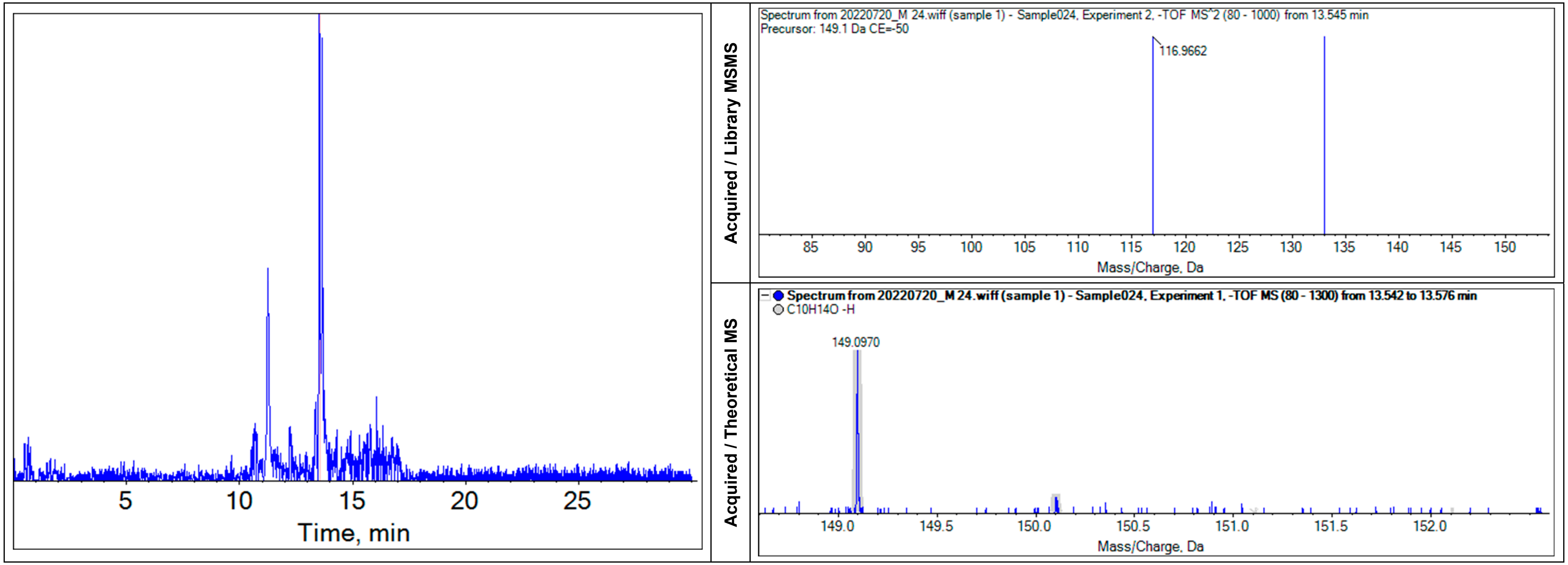

(D)

Retention Time: 13.42 minutes	Exp RT: 0.00 minutes
Extraction Mass: 149.10	Analyte Name:
Fit (%) N/A RFit (%) N/A	Thymol
	**Collision Energy = 35 ± 15 eV**

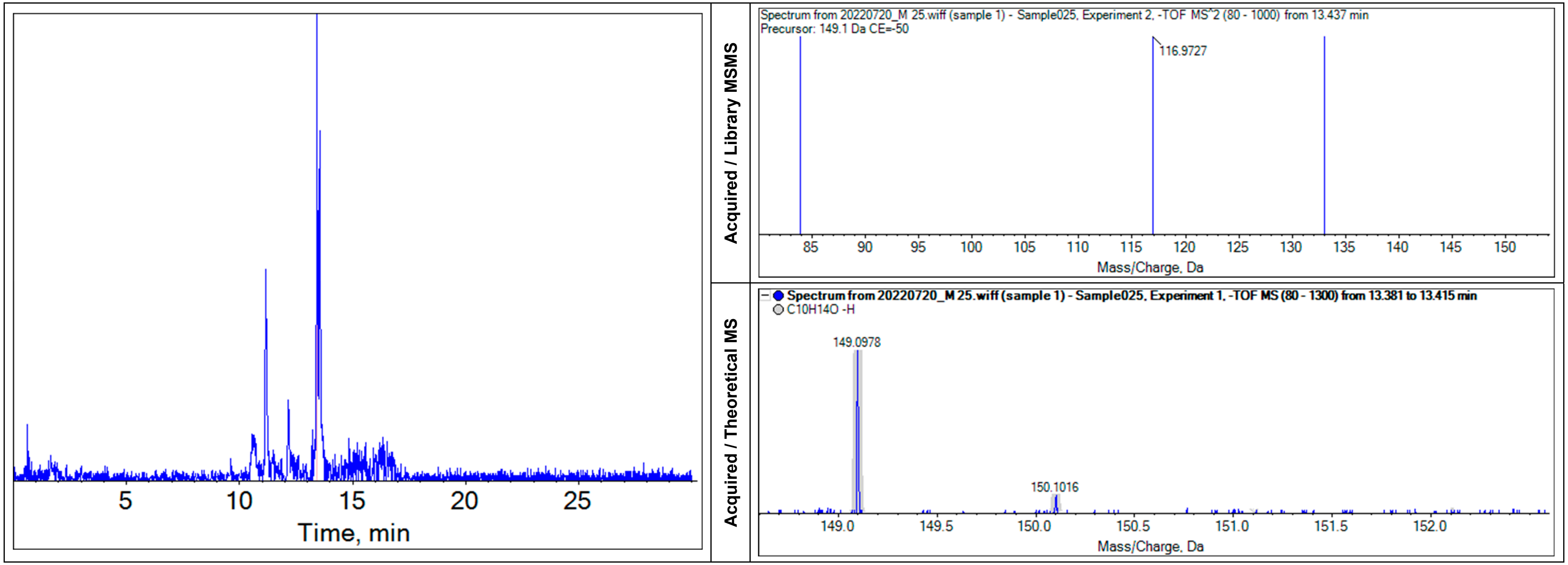

(E)

Retention Time: 13.67 minutes	Exp RT: 0.00 minutes
Extraction Mass: 149.10	Analyte Name:
Fit (%) N/A RFit (%) N/A	Thymol
	**Collision Energy = 35 ± 15 eV**

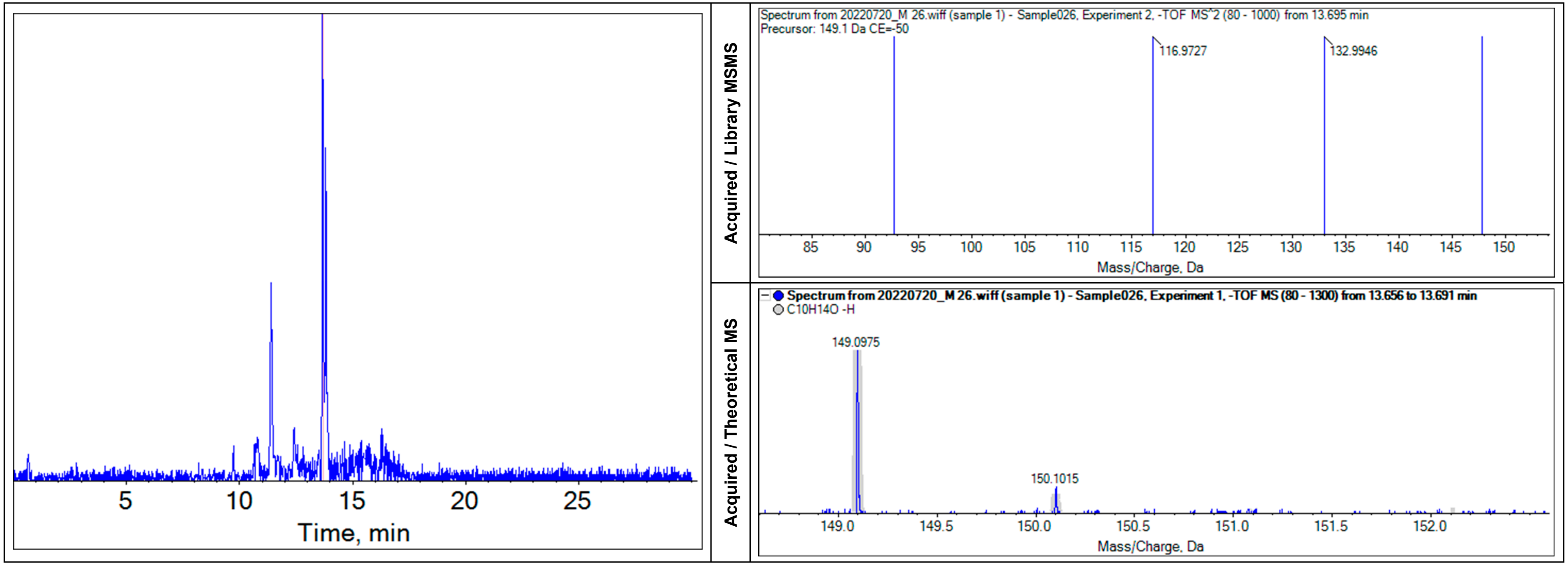

(F)

Retention Time: 13.33 minutes	Exp RT: 0.00 minutes
Extraction Mass: 149.10	Analyte Name:
Fit (%) N/A RFit (%) N/A	Thymol
	**Collision Energy = 35 ± 15 eV**

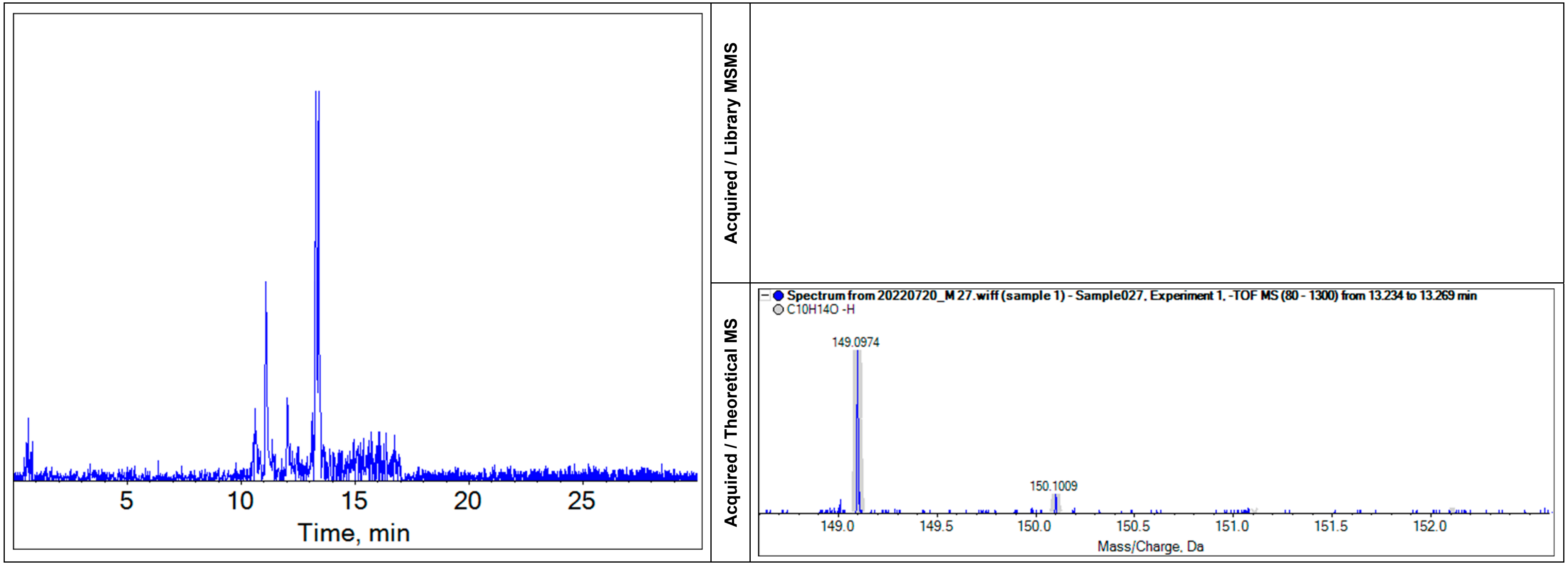

(G)

Figure S3. LC- MS/MS thymol chromatographs from (**A**) *A. brunnescens* conventional extract, (**B**) *A. brunnescens* PEF extract, (**C**) *P. ostreatus* conventional extract, (**D**) *A. bisporus* conventional extract, (**E**) *A. bisporus* PEF extract, (**F**) *L. edodes* conventional extract, (**G**) *L. edodes* PEF extract.

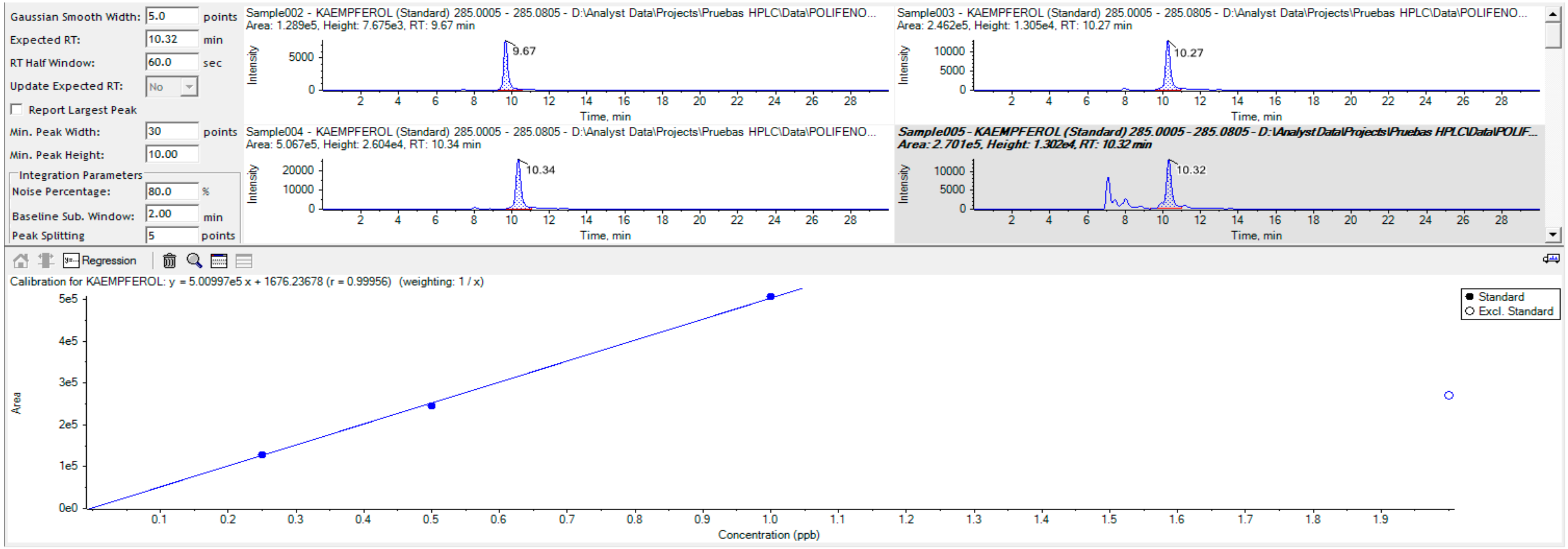
Gaussian Smooth Width: 5.0 points
Expected RT: 10.32 min
RT Half Window: 60.0 sec
Update Expected RT: No
Report Largest Peak
Min. Peak Width: 30 points
Min. Peak Height: 10.00
Integration Parameters
Noise Percentage: 80.0 %
Baseline Sub. Window: 2.00 min
Peak Splitting 5 points
Sample002 - KAEMPFEROL (Standard) 285.0005 - 285.0805 - D:\Analyst Data\Projects\Pruebas HPLC\Data\POLIFENO...
Area: 1.289e5, Height: 7.675e3, RT: 9.67 min
Sample003 - KAEMPFEROL (Standard) 285.0005 - 285.0805 - D:\Analyst Data\Projects\Pruebas HPLC\Data\POLIFENO...
Area: 2.462e5, Height: 1.305e4, RT: 10.27 min
Sample004 - KAEMPFEROL (Standard) 285.0005 - 285.0805 - D:\Analyst Data\Projects\Pruebas HPLC\Data\POLIFENO...
Area: 5.067e5, Height: 2.604e4, RT: 10.34 min
Sample005 - KAEMPFEROL (Standard) 285.0005 - 285.0805 - D:\Analyst Data\Projects\Pruebas HPLC\Data\POLIF...
Area: 2.701e5, Height: 1.302e4, RT: 10.32 min
Intensity
Time, min
Regression
Calibration for KAEMPFEROL: y = 5.00997e5 x + 1676.23678 (r = 0.99956) (weighting: 1 / x)
Standard
Excl. Standard
Area
Concentration (ppb)

(A)

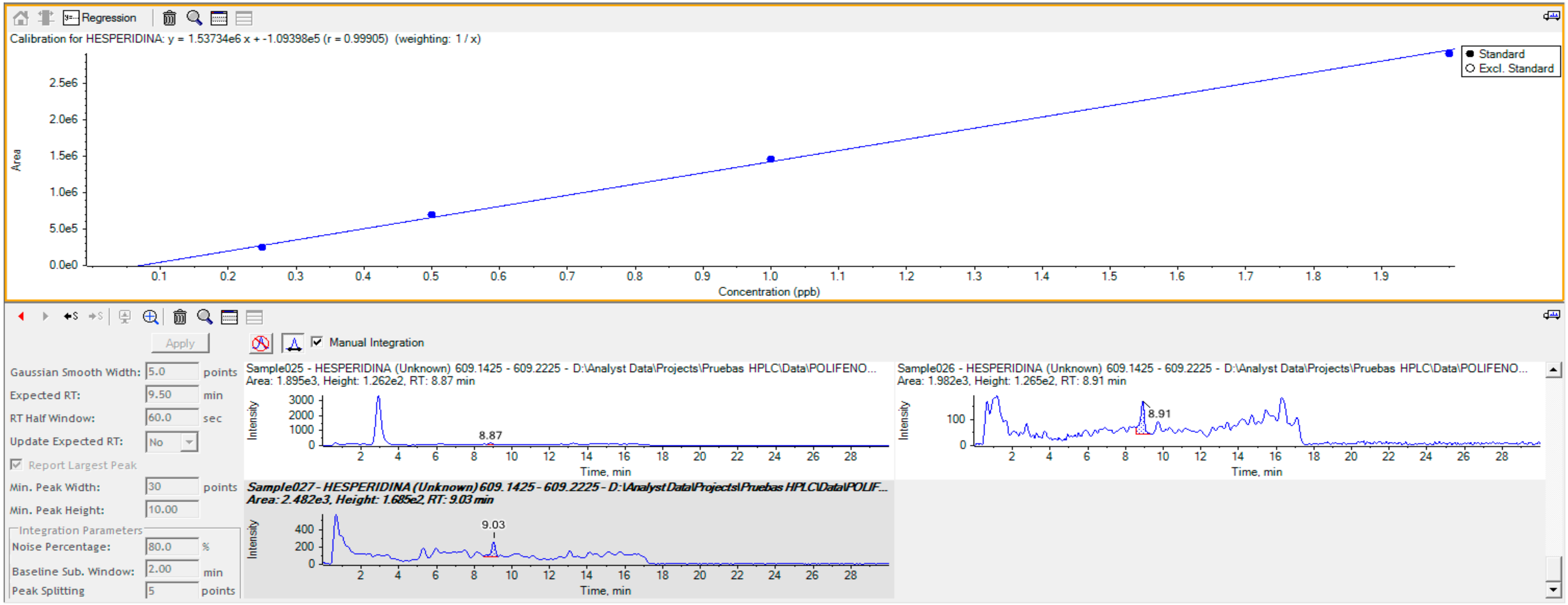

(B)

Figure S4. LC- MS/MS standard calibration of (**A**) Kaempferol and (**B**) Hesperidin with sample data showed below.

Author Contributions: Conceptualization, Mara Calleja-Gómez, Patricia Roig, Suzana Rimac-Brnčić, Francisco J. Barba and Juan Manuel Castagnini; Data curation, Mara Calleja-Gómez, Patricia Roig and Juan Manuel Castagnini; Formal analysis, Mara Calleja-Gómez and Patricia Roig; Funding acquisition, Francisco J. Barba; Investigation, Mara Calleja-Gómez; Methodology, Patricia Roig, Suzana Rimac-Brnčić, Francisco J. Barba and Juan Manuel Castagnini; Project administration, Francisco J. Barba; Resources, Francisco J. Barba and Juan Manuel Castagnini; Supervision, Francisco J. Barba and Juan Manuel Castagnini; Validation, Patricia Roig, Suzana Rimac-Brnčić, Francisco J. Barba and Juan Manuel Castagnini; Visualization, Patricia Roig, Suzana Rimac-Brnčić and Juan Manuel Castagnini; Writing – original draft, Mara Calleja-Gómez; Writing – review & editing, Mara Calleja-Gómez, Patricia Roig, Suzana Rimac-Brnčić, Francisco J. Barba and Juan Manuel Castagnini.

Funding: This research was supported by the Spanish Ministry of Science and Innovation project (PID2021-123628OB-C42 - Eco-innovative extraction of nutrients and bioactive compounds from agri-food co-products for the design of healthier foods. Study of biological activities), which is funded by MCIN/AEI/10.13039/501100011033/ and FEDER, UE.

Institutional Review Board Statement: Not applicable.

Informed Consent Statement: Not applicable.

Data Availability Statement: The data presented in this study are available on request from the corresponding author.

Acknowledgments: Juan Manuel Castagnini is beneficiary of the grant (ZA21-028) for the requalification of the Spanish university system from the Ministry of Universities of the Government of Spain, modality "Maria Zambrano", financed by the European Union, NextGeneration EU through the project "Extraction of bioactive compounds from food matrices using innovative and sustainable technologies (EXTRABIO)". The authors would also like to thank the project OTR2022-22807INVES as well as the help provided by the Laboratories of Central Support Service for Experimental Research (SCSIE)— University of Valencia for technical support in LC / MS / MS analysis for phenolic profile determination and SEM microscopy.

Conflicts of Interest: The authors declare no conflict of interest.
